# Optimizing Gas Composition and Moisture Content for Preservation of Specific Metabolites of Cultivated Stinging Nettle (*Urtica dioica* L.) Leaves

**DOI:** 10.3390/foods15101731

**Published:** 2026-05-14

**Authors:** Mia Dujmović, Mia Kurek, Sandra Voća, Nevena Opačić, Sanja Radman, Zdenko Mlinar, Jana Šic Žlabur

**Affiliations:** 1 Faculty of Agriculture, University of Zagreb, Svetošimunska Cesta 25, 10000 Zagreb, Croatia; mdujmovic@agr.hr (M.D.); svoca@agr.hr (S.V.); nopacic@agr.hr (N.O.); sradman@agr.hr (S.R.); jszlabur@agr.hr (J.Š.Ž.); 2 Faculty of Food Technology and Biotechnology, University of Zagreb, Pierottijeva 6, 10000 Zagreb, Croatia; 3 Teaching Institute of Public Health “Dr. Andrija Štampar”, Mirogojska Cesta 16, 10000 Zagreb, Croatia; zdenko.mlinar@stampar.hr

**Keywords:** leafy vegetables, postharvest quality, modified atmosphere packaging (MAP), moisture absorbers, specialized metabolites

## Abstract

The objective of this study was to evaluate the influence of modified atmosphere and moisture absorbers on specific metabolites (SMs) content in packaged stinging nettle leaves (*Urtica dioica* L.). Hydroponically cultivated fresh nettle leaves were packaged in two experiments: 1. packaging in ambient or modified atmosphere (MAP) and 2. packaging in MAP without or with moisture absorbers. The results demonstrated that both modified atmosphere and moisture absorbers statistically significantly affected (at *p* ≤ 0.05) the SM content of nettle leaves, but with opposing effects. Specifically, leaves packed in modified atmosphere had significantly higher values of phenolic compounds, ascorbic acid, total chlorophylls, and antioxidant capacity compared to ambient packaging. In contrast, the inclusion of moisture absorbers in packages was associated with a general decline in metabolite content. The highest levels of caffeoylmalic acid (405.73 mg/100 g fm), total phenolic compounds (627.35 mg GAE/100 g fm), total chlorophylls (0.87 mg/g fm), and antioxidant capacity (ABTS: 24.5 µmol TE/g, DPPH: 6.98 µmol TE/g, FRAP: 43.85 µmol TE/g fm) were observed in samples stored for 17 days under modified atmosphere without the addition of absorbers. Based on these findings, for optimal preservation of SMs during extended storage (up to 20 days), packaging stinging nettle leaves in a modified atmosphere without moisture absorbers is recommended.

## 1. Introduction

As global interest in diverse and nutrient-rich diets continues to grow, so does the demand for novel leafy vegetables that offer unique flavors, textures, and health benefits. To meet this demand, cultivation in controlled conditions, such as greenhouses or hydroponics, provides favorable conditions for introducing new leafy species, as precise regulation of environmental factors and nutrients ensures consistent growth while enhancing their nutritional profiles [1,2,3]. However, the successful commercialization of these emerging species depends not only on their cultivation but also on efficient postharvest handling and packaging strategies. Moreover, targeted packaging practices can also maintain the concentration of health-promoting compounds, ensuring the functional value and health effects of the produce. Previous research on the packaging of fresh leafy vegetables provides a useful methodological framework for studying new leafy species.

Packaging leafy vegetables ensures their freshness, nutritional quality and extends shelf life by protecting them from physical damage and microbial contamination [4,5,6,7]. However, the packaging of these vegetables presents unique challenges due to ongoing physiological processes of respiration and transpiration, which continue even after harvest and significantly influence the internal gas composition and moisture content of packaged produce. Leafy vegetables respire by consuming oxygen (O_2_) and releasing carbon dioxide (CO_2_), water vapor and heat [8]. This process involves the breakdown of carbohydrates, acids, and other nutrients in the produce to generate energy [8,9] which leads to a loss of nutritional value and sensory degradation. Simultaneously, although at a slower rate than in growing plants, water loss from leaf surfaces (transpiration) continues after harvest causing wilting and textural deterioration [10,11]. These processes are especially pronounced in leaves due to their high surface-area-to-volume ratio causing quality loss, spoilage, and shortened shelf life, so they must be carefully controlled. To mitigate these challenges, it is essential to employ appropriate storage and packaging techniques that regulate internal gas composition and humidity. Strategies such as modified atmosphere packaging (MAP), moisture absorbers, breathable packaging materials, and cold storage are commonly used to reduce respiration rates, maintain the right humidity levels, prevent microbial growth, and extend shelf life [9,12,13]. MAP is a widely used technology for preserving the freshness and quality of perishable produce by adjusting gas compositions inside the package to slow down respiration, deterioration, microbial growth and to preserve nutrients [14]. Additionally, moisture absorbers, such as silica gel packets, can help control internal humidity levels and prevent condensation, which is considered one of the greatest challenges in vegetable packaging [12,13,15,16]. Packaging in a modified atmosphere and protection against moisture accumulation requires the use of specific packaging materials with particular gas permeability and water vapor transmission properties, such as polyethylene (PE) [6,7,17,18]. Extensive studies have been conducted on the use of modified atmosphere and humidity control to extend the shelf life of common vegetables and fruits [17,19,20], but the preservation of functional properties and specific bioactive chemical compounds by modified atmosphere and moisture absorbers in newly introduced leafy species remains largely unexplored.

Plant-specific or specialized metabolites (SMs: phenolic compounds, vitamins, pigments, alkaloids, terpenoids) are organic compounds that are very important in human nutrition, as they play a key role in disease prevention contributing to health by acting as antioxidant [21], anti-inflammatory [22], and antimicrobial agents [23]. Stinging nettle (*Urtica dioica* L.) has gained attention as a medicinal plant with great health-promoting potential and has been intensively researched in the scientific community [24,25,26,27,28]. Its leaves are known for their nutritional value since they are rich in minerals, fatty acids, and SMs such as vitamins, phenolic compounds, and pigments [24,25,26,27,28], so nettle can be considered a highly nutritious and functional food. Due to its rich chemical composition and spinach-like flavor, wild harvested fresh nettle leaves are used as a green leafy vegetable in cooked dishes, smoothies, and water infusions [1].

Despite its nutritional value and culinary potential nettle has neither been systematically cultivated nor widely commercialized as a fresh leafy vegetable. Available studies have primarily focused on wild-harvested material [1,24,25,26], while information on cultivated nettle remains scarce. Cultivation, however, ensures consistency and reproducibility of plant material, making it more suitable for packaging processes. Although MAP has been extensively studied in commonly consumed leafy vegetables, little attention has been given to introduced wild medicinal species like stinging nettle, particularly in the context of postharvest handling and packaging. Furthermore, although moisture control and the use of absorbers have been investigated in relation to condensation, wilting, decay, and microbial growth in leafy vegetables, there is a lack of data on their influence on the stability of SMs. This research addresses that gap by applying moisture management strategies to nettle, a plant with distinct morphological and textural characteristics. The novelty of this work also lies in its focus on preserving metabolites responsible for the bioactive and functional properties of nettle. By integrating MAP with moisture control strategies and using cultivated nettle as a standardized raw material, this study contributes to a better understanding of how packaging conditions influence the nutritional and functional quality of nettle leaves during storage, thereby positioning nettle as a novel fresh packaged leafy vegetable. Therefore, the present study aimed to investigate whether active MAP and moisture absorbers can preserve SMs of nettle over an extended 20-day storage period. This comprehensive research includes an interdisciplinary approach from the use of cultivated plant material of known origin to the final food product by combining modern agronomic cultivation techniques with food-technological packaging solutions. Given that both atmospheric composition and moisture levels can significantly impact organoleptic properties and shelf life, the hypothesis was that these factors may also affect the chemical composition of the raw material. So, by employing these strategies the nutritional and health effects of nettle might also be maintained. The findings are expected to be relevant for both the food industry and the scientific community, as they support the development of a new marketable product and provide a foundation for future research on advanced packaging methods and the preservation of biologically active compounds in nettle leaves.

## 2. Materials and Methods

### 2.1. Experiment Setup

Stinging nettle was grown at the Department of Vegetable Crops, packaging was carried out in the Laboratory for Food Packaging, and chemical analyses were performed in the Laboratory for Quality Analysis of Agricultural Products of Plant Origin. To ensure plant material of controlled quality and origin, the cultivated nettle was used for packaging. Nettle was grown in a greenhouse during the spring–summer period of 2023 using a hydroponic technique with an ebb and flow system. Nettle seeds (B&T World Seeds, Aigues-Vives, France) were sown in polystyrene containers filled with a commercial substrate (Klasmann Potgrond H, Geeste, Germany) and nutrient solution containing a combination of salts suitable for growing leafy vegetables was used according to Dujmović et al. [29]. During the entire cultivation period, the abiotic factors of the greenhouse (air temperature and relative air humidity) and nutrient solution (pH, electrical conductivity and dissolved oxygen) were controlled. Fresh nettle leaves were collected before the flowering phase, and 30 ± 1 g was placed in commercially available polypropylene containers and packed in low-density polyethylene (LDPE) foils (Helana-pak d.o.o., Zagreb, Croatia) following three packaging treatments:

(1) Packaging under ambient atmospheric conditions (21% O_2_, 0.04% CO_2_ and 78% N_2_);
(2) Packaging in a modified atmosphere (MAP), with a gas content of 5% O_2_/5% CO_2_ and the rest nitrogen;
(3) Packaging as described in (2) but with the inclusion of the moisture-absorbing sachets (Micro-Pak Dri Clay^®^ Kraft, Micro-Pak Europe, Monte Urano, Italy).

Closing of pouches in MAP was carried out using the MAP device (WITT-Gasetechnik GmbH & Co, KM 20-3, Dorado, Junior Digit, Witten, Germany). All packages were stored for 20 days in a climate chamber (KK 750 SmartPro—PolEko, Richmond Scientific Ltd., Chorley, UK) under controlled conditions (4 °C and 80% relative air humidity). General storage parameters used in this study (temperature, relative humidity, storage duration and gas composition) were consistent with those commonly applied to leafy vegetables [4,5,6,7,13,14,16,20], with an extended storage period considering the adaptability of nettle, as well as the morphology and texture of its leaves [1]. Chemical analyses were performed on the 14th, 17th, and 20th days of storage. The research was set up as 2 two-factorial experiments with all packages and analyses performed in three repetitions.

### 2.2. Package Headspace Gas Analysis

Headspace gas analysis was performed using a portable hand-held headspace gas analyzer designed for measuring the modified atmosphere of packaged food (OXYBABY WITT-Gasetechnik GmbH & Co, KG, Witten, Germany). Changes of O_2_ and CO_2_ were measured on the day of analysis, before opening the packages. For each sampling time new packages were opened. Gas samples from the package headspace were taken by an integrated micro gas pump after needle insertion, and the concentration values were read directly on the LCD display.

### 2.3. Determination of Water and Specific Metabolite Contents

In this study well-established methods and protocols were used and are therefore briefly described, with any changes or modifications specifically emphasized. All assays are described in detail in the study by Dujmović et al. [29].

Approximately 3 g of nettle leaves were finely chopped, placed in glass jars, and dried in a hot air-drying oven (Inko, ST10EN, Zagreb, Croatia) at 105 °C until the constant weight was reached, according to AOAC [30], to determine the water content (WC). Water content was calculated as a percentage using the equation:

(1) WC (%) = (m_1_ − m_2/_m_1_ − m_0_) × 100

where m_0_ (g) is the mass of glassware; m_1_ (g) is the mass of glassware with the leaf samples before drying; m_2_ (g) is the mass of glassware with the leaf samples after drying.

For the determination of individual phenols, 1 ± 0.01 g of leaves was homogenized with 10 mL of 80% MeOH (*v*/*v*) and extracted in an ultrasonic bath (RK 103 H, Bandelin electronic, Berlin, Germany) for 30 min. Major nettle’s phenolic compounds (mg/100 g of fresh mass (fm)) were determined by reverse-phase high-performance liquid chromatography (RP-HPLC) using the non-polar (hydrophobic) stationary phase (C18 column) and polar mobile phases (A: H_2_O with 3% formic acid, and B: ACN with 3% formic acid). An LC Nexera system (Shimadzu, Kyoto, Japan) equipped with a photodiode array detector SPD-M40 and an RF-20Axs fluorescence detector, a DGU-405 degasser, an LC-40B XR pump, a CTO-40C column oven, and an SIL-40C autosampler was used.

Preparation of plant material for analyses of the total phenols and antioxidant capacity was the same; 10 ± 0.01 g of leaves were boiled with 100 mL of 80% EtOH (*v*/*v*). Total phenolic (mg GAE/100 g fm), total flavonoid (mg CTH/100 g fm) and total non-flavonoid compounds (mg GAE/100 g fm) were detected with the Folin–Ciocalteu reagent method and the selective precipitation method according to Ough and Amerine [31] and Dujmović et al. [29]. Antioxidant capacity was evaluated by three methods: DPPH radical scavenging activity, ABTS^●+^ radical scavenging activity, and ferric ion reducing antioxidant power (FRAP) [28]. For the determination of photosynthetic pigments (chlorophyll a, chlorophyll b, total chlorophylls and total carotenoids) leaves were homogenized in acetone (p.a.) and filtered and determined according to Holm [32] and von Wettstein [33]. Spectrophotometric analyses of total phenolic compounds, antioxidant capacity and photosynthetic pigments were performed on a 1900i spectrophotometer (Shimadzu, Kyoto, Japan).

Shredded plant material was extracted for several minutes in 100 mL of 2% (*v*/*v*) oxalic acid, for the detection of ascorbic acid (AsA), and its content (mg/100 g fm) was determined by titration with 2,6-dichlorophenolindophenol using the AOAC method [30]. All chemical compounds were analyzed in triplicate.

### 2.4. Statistical Analyses

Prior to analysis statistical test’s assumptions (normality of residuals, homogeneity of variance and absence of outliers) were visually verified using Q-Q plots. All datasets met the assumptions required for analysis of variance (ANOVA). The data were processed by two-way ANOVA which was performed using the SAS program with GLM procedure, v 9.4. Means were compared by LSD *t*-test and differences were considered significant at *p* ≤ 0.05. Statistical differences among treatments are indicated in the figures by letter annotations. Statistical conclusions were based on ANOVA results, post hoc LSD grouping, and associated probability (*p*) values.

Two experiments were conducted as part of this research. In each experiment, the effects of two factors on the content of a certain dependent variable were evaluated:

1. Type of atmosphere and storage duration (AT × D);
2. Moisture absorber and storage duration (A × D).

## 3. Results and Discussion

### 3.1. Gas Composition in Packages During Storage

**Figure 1:**
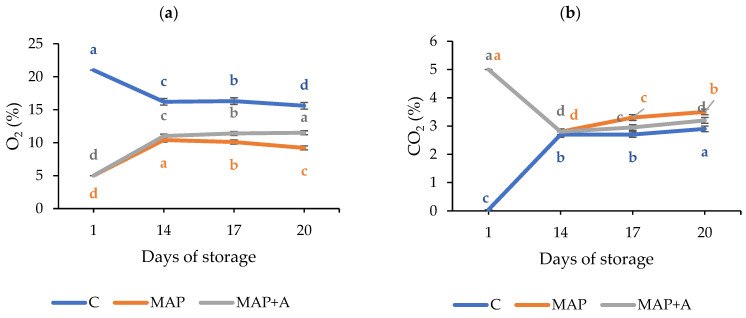
Changes in O_2_ (**a**) and CO_2_ (**b**) levels in the package headspace during the 20-day storage period of fresh nettle leaves. Samples: C—leaves packaged in ambient atmosphere with no gas modification, MAP—leaves packaged with active modified atmosphere (5% O_2_/5% CO_2_), MAP+A—leaves packaged with active modified atmosphere and moisture absorber. Different letters (a–d) denote statistically significant differences (*p* < 0.05).

During storage, changes in the gas content of the packages were monitored. The gas composition of nettle samples packaged in ambient conditions (C), modified atmosphere (MAP) and modified atmosphere with moisture absorbers (MAP+A) is given in Figure 1a,b. Statistically significant differences in the composition of both gases between storage days (1, 14, 17 and 20 days) were observed in all samples. Also, the measurements clearly show the difference between the gas composition in packages with modified atmosphere (MAP and MAP+A) and ambient atmosphere (C). In the C samples, the O_2_ concentration decreased from 21% to 15.6% after 20 days, while CO_2_ increased from ambient levels (0.04%) to 2.9%. Respiration-driven changes in gas composition in polymer-packaged plant products imply the development of a passive modified atmosphere and have been previously observed in leafy vegetables such as spinach [34,35], lettuce [36,37], and mulberry leaves [38]. In MAP samples, the initial O_2_ concentration of 5% increased to 9.2% by day 20, while CO_2_ reduced from 5% to 3.5%. A similar pattern was observed in MAP+A, where O_2_ increased to 11.5% and CO_2_ decreased to 3.2%, showing that the presence of moisture absorbers (MAP+A) caused a slight increase in O_2_ content and a slight decrease in CO_2_ compared to the MAP samples. After 14 days, an equilibrium state was reached in all samples. It can be noticed that gas composition in the active modified atmosphere packages deviated from the initial conditions (5% O_2_ and 5% CO_2_) during storage, with an unexpected increase in O_2_ and a decrease in CO_2_ content observed between days 1 and 14. However, after 20 days of storage, the atmosphere remained significantly different from both ambient air and C samples, particularly for O_2_. In fact, during the entire storage period, the MAP samples showed the lowest O_2_ levels and the highest CO_2_ content, while the opposite was detected in the C samples. These gas fluctuations observed in nettle packages are similar to those previously reported for leafy vegetables stored under MAP [20,34,35,36,39,40]. Furthermore, in the present study, the initial modified atmosphere used was consistent with previous studies on leafy vegetable packaging, which recommend initial modified gas compositions of 1–7% O_2_ and 3–17% CO_2_ [7,20,28,34,38,41,42,43,44,45,46]. In vegetable packaging, both excessively low O_2_ levels and high CO_2_ concentrations are undesirable, as rapid O_2_ depletion may induce anaerobic metabolism [47], while excessive CO_2_ accumulation can result in off-flavors or tissue damage [5,48]. In this study, in MAP packed samples, levels of O_2_ were between 5 and 11.5% and CO_2_ remained within a range considered optimal and beneficial for leafy vegetables (2.8–5%). Authors Al-Ati and Hotchkiss [49] highlighted the role of the produce’s respiration rate, packaging film permeability and thickness, and storage temperature in maintaining modified atmosphere and product quality. In this context, the main reason for the change in gas composition in closed packages may be attributed to leaf respiration and the selective gas permeability of the polyethylene film (as seen in Dujmović et al. [27]). Generally, packaged leafy vegetables consume O_2_ and release CO_2_ within the package, while gas exchange with the external atmosphere occurs due to the permeability of the packaging material [34,35,36,37,38]. Although an unexpected change in gas composition was observed by the 14th day, MAP samples consistently exhibited the lowest O_2_ and highest CO_2_ levels over the entire storage period, indicating the effectiveness of the modified atmosphere in maintaining these conditions during 20 days of nettle storage in LDPE pouches. Reduced O_2_ levels are known to slow down respiration, enzymatic processes, and metabolite degradation [14,50], while moderate CO_2_ levels inhibit microbial growth and oxidative enzyme activity [5,51,52], together contributing to improved stability of plant material during storage. Therefore, these conditions may also be beneficial for the preservation of SMs. Although respiration rate and enzyme activity were not directly measured in this study, the observed gas composition dynamics are consistent with established postharvest physiological responses reported in the literature.

### 3.2. Chemical Composition and Antioxidant Capacity of Packaged Nettle Leaves

The significance of the influence of tested individual factors and their interaction on the content of water, specific metabolites and antioxidant capacity of packaged nettle leaves is given in the Supplementary Materials in Table S3a,b.

#### 3.2.1. Water Content

**Figure 2:**
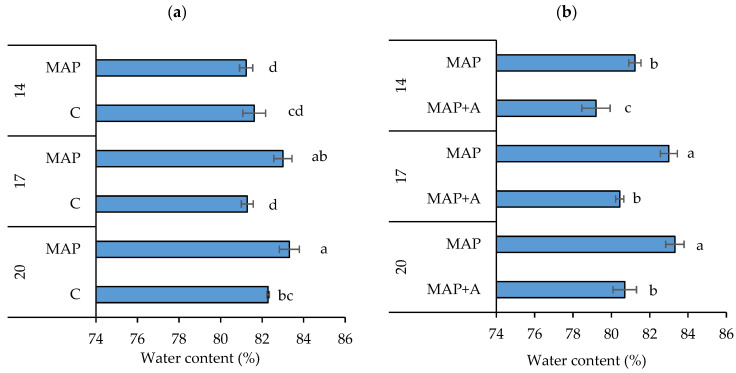
The water content (WC, %) in fresh nettle leaves stored for 20 days and packaged in (**a**) modified and ambient atmosphere, (**b**) modified atmosphere with and without moisture absorbers. Means ± SD followed by the same letters are not significantly different at *p* ≤ 0.05 by LSD test (n = 3). Samples: C—leaves packaged in ambient atmosphere with no gas modification, MAP—leaves packaged with active modified atmosphere (5% O_2_/5% CO_2_), MAP+A—leaves packaged with active modified atmosphere and moisture absorber.

The effect of MAP on the water content (WC) of fresh nettle leaves stored for 20 days is given in Figure 2a. A significant interaction of factors, i.e., atmosphere type and days of storage (AT × D, *p* = 0.0025), was observed showing an influence of MAP on WC during storage (Table S3a). After 14 days of storage, there was no significant difference between the water content in leaves packaged in ambient atmosphere (C, 81.62%) and leaves packed in MAP (81.23%). However, as storage time increased to 17 and 20 days, the water content in MAP samples (83% and 83.31%, respectively) surpassed that of C samples (81.28% and 82.29%, respectively), being significantly higher. These findings are consistent with previous studies showing that MAP can reduce moisture loss and maintain water content in packaged fresh fruits and vegetables [53,54,55,56,57,58]. By minimizing moisture loss MAP helps in maintaining turgidity and freshness while slowing quality degradation and consequently extending product shelf life [54]. Cefola et al. [55] reported that storing table grapes in MAP increased the relative water content of peduncles. The effect of a modified atmosphere on the moisture content of leafy vegetables is even more pronounced because the leaves are covered with stomata through which gas exchange and water vapor transpiration take place [11,59]. Stomata typically close in response to high CO_2_ concentrations [60], so elevated CO_2_ levels in MAP create an environment that minimizes water loss by controlling respiration and transpiration rates. It is noteworthy that the effect of MAP in our study became more pronounced at 17 and 20 days, indicating that the influence of MAP on water retention became more effective as storage time increased. The increase in WC under MAP conditions suggests that this packaging method is particularly effective in preserving the freshness of nettle leaves. Although these effects are beneficial, too much water can lead to rotting and the development of microorganisms [61], so moisture levels should be regularly monitored.

Figure 2b demonstrates the effect of moisture absorbers within MAP during storage on the water content (WC) of packaged fresh nettle leaves. According to statistical analyses in Table S3b, both individual factors (A and D) had a significant influence on WC, but their interaction (A × D) had no effect on WC in nettle leaves. Results show that the presence of moisture absorbers had a significant impact on water retention, particularly at longer storage durations. On the 14th day of storage, the WC in leaves packaged in MAP without moisture absorbers (MAP, 81.23%) was significantly higher than in leaves packaged with moisture absorbers (MAP+A, 79.20%). This trend continued after 17 and 20 days, where MAP samples consistently maintained significantly higher WC (83% and 83.31%, respectively) compared to MAP+A (80.44% and 80.70%, respectively). The difference in WC between MAP+A and MAP samples was more pronounced at longer storage (17 and 20 days), indicating that the effects of moisture absorbers became more evident over time. The lower WC in samples with absorbers is expected due to their role in actively removing excess moisture from the packaging environment. Although these effects are generally desirable for packaged vegetables, excessive dehydration can induce osmotic stress, resulting in loss of cell turgor and disruption of membrane integrity. However, although statistically significant differences were observed, the typical water content of nettle was not impaired. Moreover, after 20 days of storage, MAP+A samples still contained water content that is considered characteristic for fresh nettle leaves (79.20–83.31%) [27,62,63]. Hence, even after such an extended period, which the literature considers long for fresh leafy vegetables [64,65], there was no excessive dehydration of samples packed in MAP with moisture absorbers.

According to several studies [15,16], one of the key challenges in vegetable packaging is the formation of condensation inside the packaging, often caused by temperature fluctuations during transport and storage. This excess moisture, released from plants through transpiration and respiration, creates ideal conditions for the growth of mold, bacteria, and other pathogens, compromising product safety and quality [12,61]. Therefore, actively lowering water content can help inhibit microbial growth, extend shelf life, and reduce food waste. But it should be noted that both too high or too low WC in produce and inside the packaging are undesirable, as they can lead to rotting or dehydration.

#### 3.2.2. Specific Metabolites

##### Phenolic Compounds

**Figure 3:**
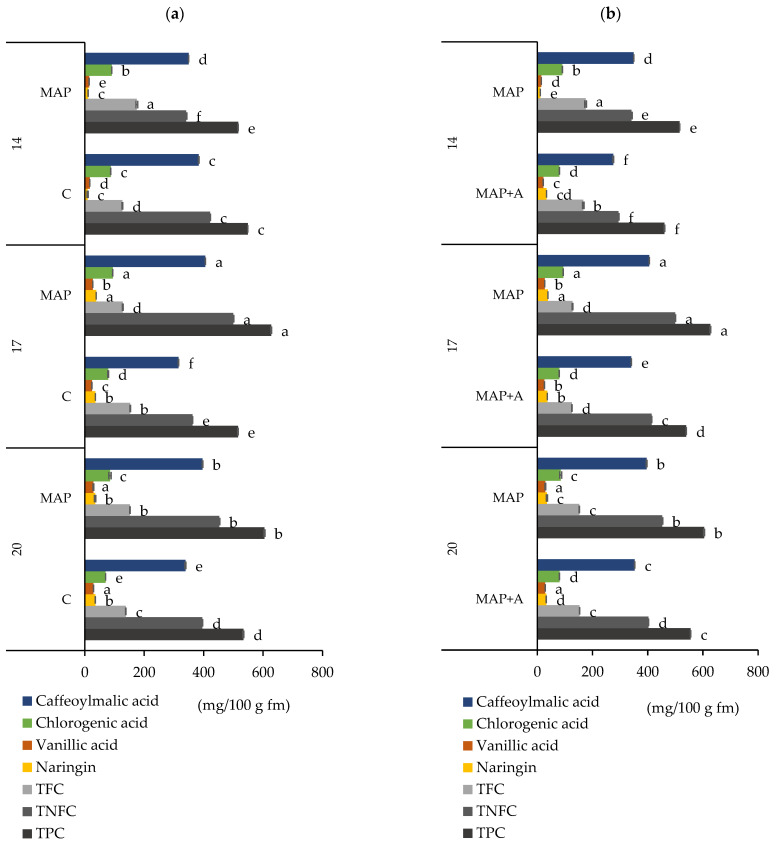
The content of individual phenolic compounds (caffeoylmalic acid, chlorogenic acid, vanillic acid and naringin, mg/100 g) and total phenolic compounds (TFC—total flavonoid compounds, mg CTH/100 g fm, TNFC—total non-flavonoid compounds, mg GAE/100 g fm, TPC—total phenolic compounds, mg GAE/100 g fm) in fresh nettle leaves stored for 20 days and packaged in (**a**) modified and ambient atmosphere, (**b**) modified atmosphere with and without moisture absorbers. Means ± SD followed by the same letters are not significantly different at *p* ≤ 0.05 by LSD test (n = 3). Letters indicate differences in the content of each component separately. Samples: C—leaves packaged in ambient atmosphere with no gas modification, MAP—leaves packaged with active modified atmosphere (5% O_2_/5% CO_2_), MAP+A—leaves packaged with active modified atmosphere and moisture absorber.

Total phenolic content (TPC) and selected individual phenolic compounds in packaged nettle leaves were investigated, and the results are shown in Figure 3a,b. Previous studies have identified caffeoylmalic acid as the main individual phenolic compound in nettle leaves, followed by chlorogenic acid, with these two compounds contributing most to the biological and functional properties of nettle [27,28,66,67,68,69]. Other phenolic compounds frequently found in nettle, but at lower levels, include some flavonoids such as kaempferol, quercetin, rutin, and isorhamnetin as well as phenolic acids such as caffeic acid, *p*-coumaric acid and ferulic acid [26,70,71]. In the present research, a similar phenolic profile was found, with significant amounts of caffeoylmalic, chlorogenic, and vanillic acid and naringin, while other compounds were detected at noticeably lower concentrations. Based on their quantitative dominance and biological relevance, the results for these four predominant phenolic compounds in fresh packaged nettle leaves were presented. Phenolic compounds detected in substantially lower concentrations were not discussed in detail to maintain focus on the most physiologically relevant metabolites but are included in Supplementary Materials (Table S4a,b).

The impact of modified atmosphere on phenolics during storage is shown in Figure 3a and Table S3a. A strong effect of factors interaction, AT × D (atmosphere type and days of storage), on caffeoylmalic, chlorogenic and vanillic acids and total phenolic compounds (TPC) was observed (Table S3a, *p* ≤ 0.0001). After 14 days, samples packed in MAP (349.38 mg/100 g) had significantly lower levels of the key phenolic compound, caffeoylmalic acid, compared to C samples (383.55 mg/100 g). However, after 17 and 20 days, this trend reversed, with leaves packaged in MAP having higher caffeoylmalic values than those in C. Chlorogenic acid levels were consistently higher in MAP samples during the storage period compared to those packaged in ambient atmosphere (C), indicating that MAP provides a stable environment for chlorogenic acid. Vanillic acid showed an interesting pattern with the same trend as caffeoylmalic acid at days 14 and 17, but with the exception at day 20, when vanillic acid levels between MAP and C packages did not statistically differ. The effect of the interaction of factors on naringin was significant, but moderate (*p* = 0.0474, Table S3a), so naringin levels were the same in MAP and C samples after 14 and 20 days, but were significantly higher in MAP samples on day 17. Although the content of compounds varied during storage, the highest levels of most of the tested individual phenolic compounds (caffeoylmalic and chlorogenic acid and naringin) were recorded after 17 days of storage in nettle leaves in MAP. Consequently, the highest level of TPC was found in the same sample (MAP at 17 days). Basically, TPC followed the same trend as caffeoylmalic acid, which was expected. After 14 days of storage, TPC values were significantly higher in C samples (548.38 mg GAE/100 g) than in MAP samples (516.00 mg GAE/100 g). However, at later storage times (days 17 and 20), TPC was significantly higher in MAP samples. Very high amounts of total phenolics were also found in samples stored for 20 days in MAP. The content of non-flavonoid compounds (TNFC) showed the same trend as TPC, with higher content in MAP samples on days 17 and 20. TNFC values were higher than TFC values in all samples, most likely because the most abundant compounds detected were phenolic acids. Total flavonoid compounds (TFC) had higher values in leaves packaged in MAP samples on day 14 and 20. The obtained results show that MAP may have initially suppressed the increase in phenolic compound content, especially caffeoylmalic acid, vanillic acid and TPC. This may reflect an initial response of the leaves to modified atmosphere conditions. Over time, however, the protective effects of MAP became more pronounced, leading to higher phenolic levels (caffeoylmalic and chlorogenic acid and TPC) at days 17 and 20 compared to non-MAP samples. These results are consistent with previous studies showing reduced degradation of phenolic compounds under MAP conditions for several leafy vegetables. For instance, Gil-Izquierdo et al. [72] reported an increase in phenolic content in the internal bracts of artichokes stored under MAP, especially when low-density polyethylene (LDPE) was used as the packaging material; while Kaur et al. [73] observed a significant increase in phenolic concentration in spinach in LDPE packages stored in low-O_2_ and high CO_2_ environments, similar to those in the present research. Additionally, Naheed et al. [74] found that increased O_2_ levels within packaging accelerate flavonoid degradation, confirming the critical relationship between O_2_ concentration and the stability of these compounds. These observations may reflect the sensitivity of phenolic compounds to environmental conditions such as changes in O_2_ concentration [75,76,77]. Furthermore, a review of the scientific literature indicates that MAP and gas composition influence phenolic compounds in plant tissues. According to previous studies, low O_2_ levels inhibit the activity of oxidative enzymes such as polyphenol oxidase (PPO) and peroxidase (POD), thereby reducing phenolic degradation [51,78]. In addition, elevated CO_2_ concentrations may suppress microbial activity and modulate key enzymes involved in phenolic metabolism by inhibiting PPO [79] and stimulating phenylalanine ammonia-lyase (PAL) [80]. Reduced O_2_ combined with increased CO_2_ also slows respiration and overall metabolic activity [14]. Regardless of the mechanisms underlying the observed behavior of phenolic compounds, which were not investigated in this study, MAP was effective in maintaining phenolic content, while increases in certain compounds were also observed during storage.

The results in Table S3b show the impact of moisture absorbers on the content of individual and total phenolic compounds in fresh nettle leaves packaged in MAP during storage. Statistical analyses showed a significant influence of the interaction of factors: A × D (moisture absorbers and days of storage) on the stability of the analyzed compounds (caffeoylmalic, vanillic acid, naringin, TPC and TNFC: *p* ≤ 0.0001; chlorogenic acid: *p* = 0.0004; TFC: *p* = 0.0033). Moreover, the data in Figure 3b indicate that moisture absorbers significantly affected both individual and total phenolic compounds, with MAP+A samples generally showing lower levels of caffeoylmalic acid, chlorogenic acid, and TPC compared to MAP samples. Vanillic acid and naringin exhibited mixed responses to moisture control. For vanillic acid, on day 14 MAP+A samples (21.56 mg/100 g) showed higher levels than MAP (14.61 mg/100 g), but at longer storage periods (days 17 and 20), there was no statistical difference between MAP and MAP+A packages. Naringin levels were higher in MAP+A samples on the 14th day (33.59 mg/100 g) than in MAP samples (11.13 mg/100 g). However, this trend reversed during storage, with significantly higher naringin content in MAP samples on days 17 and 20. During the storage period, TPC and TNFC were significantly higher in leaves packaged in MAP compared to MAP+A samples, with the difference becoming more pronounced at longer storage durations (17 and 20 days). It should be noted that the moisture absorbers did not consistently reduce phenolic content during storage; however, a decreasing trend was observed for most phenolic compounds (TPC, TNFC, caffeoylmalic and chlorogenic acid). The initial hypothesis of this study was that moisture absorbers could improve postharvest quality by regulating moisture levels in the packaging system, thereby influencing the stability of phenolic compounds in high-transpiration leafy vegetables such as nettle. Given the analytical methods used in this research, the observed changes cannot be interpreted as direct mechanistic conclusions derived from the present results but should be interpreted in the context of existing scientific literature. Physiological processes that could be driven by high moisture, such as senescence, microbial decay, and spoilage, are known to contribute to the degradation of SMs [52,81]; therefore, the use of absorbers may be beneficial. Also, as widely reported for field-grown crops [82,83,84] moisture levels in plant tissues have been shown to influence phenolic stability; however, postharvest humidity effects remain less explored. It is well established that water availability strongly influences microbial activity and enzymatic reactions involved in biodegradation processes [85,86]. Dehydration, in particular, can enhance the activity of oxidative enzymes such as polyphenol oxidase (PPO) and peroxidase (POD), which catalyze the degradation of phenolic compounds [87,88]. Moreover, water loss disrupts cellular water balance, inducing osmotic stress that impairs the maintenance of turgor pressure and normal metabolic function. At the same time, dehydration alters membrane structure and fluidity, often resulting in the leakage of metabolites [82,83,84,85,86] and facilitating contact between oxidative enzymes and phenolic substrates, thereby promoting their enzymatic oxidation [87,88]. Some studies have also suggested that water loss can lead to the degradation of phenolic compounds in leaves and leafy vegetables [89,90]. Although phenolic compounds are moderately to highly water-soluble [81,91], they are not volatile and do not evaporate from tissues through transpiration. Thus, their decline cannot be explained by physical loss during moisture evaporation. In this present research moisture absorbers caused a statistically significant reduction in water content, so they likely lowered the internal water level to the extent that triggered cellular stress responses, including osmotic stress, membrane disruption and oxidative damage.

##### Ascorbic Acid

**Figure 4:**
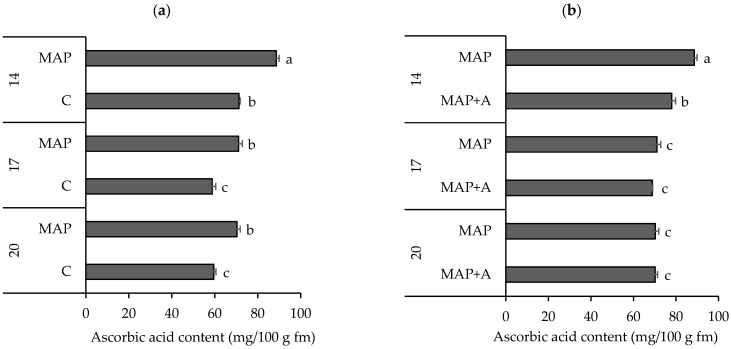
The content of ascorbic acid (AsA, mg/100 g fm) in fresh nettle leaves packaged in (**a**) modified and ambient atmosphere, (**b**) modified atmosphere with and without moisture absorbers and stored for 20 days. Means ± SD followed by the same letters are not significantly different at *p* ≤ 0.05 by LSD test (n = 3). Samples: C—leaves packaged in ambient atmosphere with no gas modification, MAP—leaves packaged with active modified atmosphere (5% O_2_/5% CO_2_), MAP+A—leaves packaged with active modified atmosphere and moisture absorber.

The influence of modified atmosphere on ascorbic acid (AsA) content and the significance of factor interactions (AT × D: *p* = 0.0022) noted in samples are given in Figure 4a and in Table S3a, respectively. The data indicate that the atmosphere type significantly affected AsA content, particularly after the 14th day. During the first 14 days of storage, the AsA content in leaves packaged with MAP (88.60 mg/100 g) was significantly higher than in C samples (71.21 mg/100 g). This trend continued at 17 and 20 days, where MAP samples consistently maintained higher AsA levels (71.07 and 70.33 mg/100 g, respectively) compared to C samples (58.80 and 59.50 mg/100 g, respectively). AsA is highly sensitive to O_2_, light, and elevated temperatures, and it degrades rapidly through oxidative reactions, particularly during postharvest handling and storage [92,93]. By altering the gaseous environment surrounding the packaged produce MAP limits the availability of O_2_. This low-oxygen environment inhibits the activity of ascorbate oxidase and other oxidative enzymes that catalyze AsA degradation [93]. Simultaneously, elevated CO_2_ levels help reduce the respiration rate of plant tissues [5,9], thereby decreasing metabolic consumption of AsA. Additionally, MAP diminishes the generation of reactive oxygen species (ROS), which are known to accelerate AsA degradation [94]. Higher AsA content under MAP observed in this study is consistent with previous findings in leafy vegetables, including lettuce, Chinese cabbage, and baby mustard, where modified atmosphere packaging maintained AsA levels [95,96,97].

The data from Figure 4b and Table S3b (A × D: *p* ≤ 0.0001) reveal that the presence of moisture absorbers in modified atmosphere packages had a significant impact on AsA content, particularly during the early stages of storage. After 14 days, leaves stored in MAP (88.60 mg/100 g) had significantly higher AsA content compared to MAP+A (77.99 mg/100 g). The lower initial values of AsA in MAP+A samples could be attributed to moisture removal, which may have disrupted the cellular structure of the leaves, leading to faster oxidation of AsA. More precisely, since AsA is a water-soluble vitamin [98], its water solubility makes it highly dependent on the plant’s water content for its accumulation and distribution. Additionally, a study by Seminario et al. [99] showed that water deficit can reduce AsA levels in leaves. However, as storage time increased to 17 and 20 days, the differences between MAP and MAP+A samples diminished, with no significant difference between them indicating that the effect of moisture absorbers on AsA becomes less pronounced over time. Findings from this study have important implications for packaging fresh nettle leaves, as the use of moisture absorbers may not be beneficial for the preservation of AsA content in nettle leaves, especially for a 14-day storage period, or may have no effect at all for a longer storage period.

##### Photosynthetic Pigments

**Figure 5:**
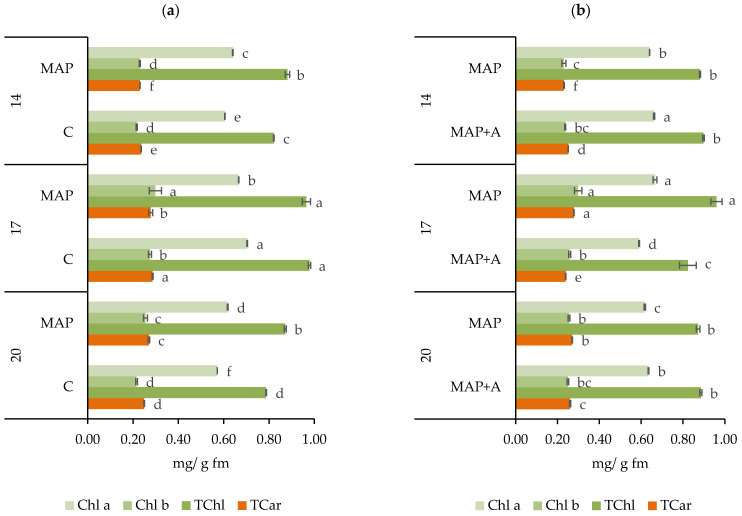
The content of chlorophyll a (Chl a), chlorophyll b (Chl b), total chlorophylls (TChl) and total carotenoids (TCar) (mg/g fm) in fresh nettle leaves packaged in (**a**) modified and ambient atmosphere, (**b**) modified atmosphere with and without moisture absorbers and stored for 20 days. Means ± SD followed by the same letters are not significantly different at *p* ≤ 0.05 by LSD test (n = 3). Letters indicate differences in the content of each component separately. Samples: C—leaves packaged in ambient atmosphere with no gas modification, MAP—leaves packaged with active modified atmosphere (5% O_2_/5% CO_2_), MAP+A—leaves packaged with active modified atmosphere and moisture absorber.

The results and statistical significance of the interaction between atmosphere type and days of storage for photosynthetic pigment content are shown in Figure 5a and Table S3a (AT × D: *p* ≤ 0.0001). The modified atmosphere significantly influenced the total photosynthetic pigments of nettle leaves over the storage period. In general, samples stored under MAP conditions maintained higher levels of chlorophyll a (Chl a), chlorophyll b (Chl b), i.e., total chlorophylls (TChl), and total carotenoids (TCar) compared to those stored in ambient atmosphere (C). On day 14, leaves packed in MAP exhibited significantly higher contents of Chl a and TChl compared to C samples. On day 17 the trend changed, and the content of TChl was the same in MAP and C samples. By day 20, MAP samples again showed significantly higher individual and total chlorophyll levels compared to C samples. As for carotenoids, they were significantly higher in C packages on days 14 and 17, but after 20 days leaves packed in MAP had statistically higher values of total TCar. Overall, the data showed that MAP maintained higher levels of photosynthetic pigments in nettle leaves, especially Chl a and TChl, after 14 and 20-day storage. These findings are consistent with studies on other leafy vegetables, where MAP has been shown to stabilize chlorophyll and carotenoid levels [35,44,100]. In general, chlorophyll degradation is a part of the aging process in leaves [101]. However, after harvest and packaging, it follows specific biochemical pathways influenced by storage conditions and enzymatic activity. Among packaging conditions, balanced gas levels, along with temperature and humidity, play a key role in maintaining chlorophyll content [20,35,100]. In modified atmosphere packaging, where leafy vegetables are stored without sunlight and photosynthesis is inactive, CO_2_ and O_2_ influence chlorophyll content through physiological and biochemical processes related to oxidation, respiration and chlorophyll catabolism [5,9,102]. Elevated CO_2_ levels help slow down leaf respiration and inhibit enzymes involved in chlorophyll degradation such as chlorophyllase [102]. As high O_2_ levels accelerate respiration, oxidative processes and chlorophyll degradation, lowering O_2_ concentrations reduce respiration rates, contributing to the maintenance of chlorophyll [9]. Carotenoids are pigments also sensitive to abiotic factors such as O_2_, light, and temperature [103]. By reducing O_2_ levels and increasing CO_2_ concentrations, MAP helps to slow down respiration, limit oxidative reactions and enzymatic activity [20] that can lead to carotenoid loss as well. The effect of MAP on carotenoids was noticeable only on day 20, with MAP samples showing higher carotenoid levels during extended storage.

The results in Figure 5b show the effect of moisture absorbers in MAP on the chlorophyll and carotenoid content of nettle leaves during storage. Statistical analysis (Table S3b) showed that moisture absorbers significantly affected photosynthetic pigments, with a significant interaction between moisture absorber and days of storage (A × D) across all pigments analyzed (Chl a: *p* ≤ 0.0001, Chl b: *p* = 0.0003, TChl: *p* ≤ 0.0009, TCar: *p* ≤ 0.0001). Chl a was significantly higher in MAP+A packages after 14 and 20 days, but after 17 days it was lower in MAP+A. Similarly, after 17 days, the Chl b value was also significantly lower in the sample with the absorber (MAP+A). No significant differences in TChl were observed between MAP and MAP+A after 14 and 20 days, whereas after 17 days significantly higher TChl levels were found in MAP samples. After 14 days, TCar were higher in MAP+A packages, but for the remaining storage period, higher values of TCar were recorded in leaves packed in MAP. Overall, the highest content of TChl and TCar was recorded after 17 days in MAP samples (with no absorbers). The effect of moisture on the content of photosynthetic pigments is not completely clear yet. It is generally understood that too high moisture can accelerate chlorophyll degradation through enzymatic and microbial activity [104,105], while excessive dryness can also negatively affect chlorophyll by causing cellular damage and oxidative and osmotic stress. Chlorophyllase, the enzyme responsible for chlorophyll degradation, exhibits increased activity under high humidity, accelerating chlorophyll breakdown [104]. Lee and Chandra [4] reported that chlorophyll degradation was slowed down in an anti-fog packed lettuce. However, the results of the present study contrast with these findings, as the reduction in moisture content, achieved using absorbers, primarily led to a decrease in photosynthetic pigment content. In conclusion, while the use of moisture absorbers may temporarily preserve or even increase pigment content, the overall impact was limited. The results suggest that modified atmosphere packaging alone is sufficient to maintain photosynthetic pigment levels, and the addition of absorbers does not provide significant added benefits.

#### 3.2.3. Antioxidant Capacity

**Figure 6:**
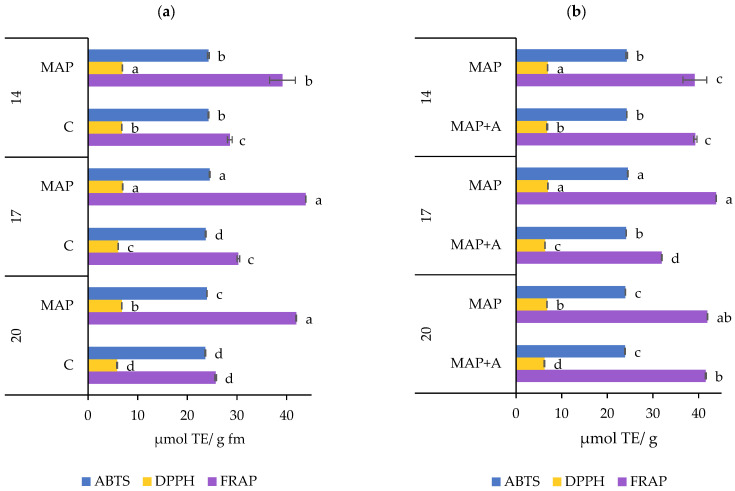
Antioxidant capacity (µmol TE/g fm) of fresh nettle leaves stored for 20 days and packaged in (**a**) modified and ambient atmosphere, (**b**) modified atmosphere with and without moisture absorbers. Means ± SD followed by the same letters are not significantly different at *p* ≤ 0.05 by LSD test (n = 3). Letters indicate differences in the content of each component separately. Samples: C—leaves packaged in ambient atmosphere with no gas modification, MAP—leaves packaged with active modified atmosphere (5% O_2_/5% CO_2_), MAP+A—leaves packaged with active modified atmosphere and moisture absorber.

The antioxidant capacity of fresh nettle leaves was measured by three methods (ABTS, DPPH, and FRAP assays) and the results are shown in Figure 6a. Statistical significances presented in Table S3a show significant interaction of type of atmosphere and days of storage (AT × D) on the antioxidant capacity (*p* ≤ 0.0001 for ABTS and DPPH, *p* = 0.0024 for FRAP). The application of MAP significantly influenced the antioxidant capacity of nettle leaves, as demonstrated by all three analytical methods used. For ABTS values, MAP and C samples differed significantly, although the absolute differences were small (23.63–24.50 µmol TE/g). After 14 days, leaves packed and stored in MAP and C were not statistically different, but after 17 and 20 days, MAP samples showed significantly higher antioxidant activity than C samples. The DPPH test showed clearer distinctions, with MAP samples exhibiting significantly higher antioxidant capacity than the corresponding ambient samples. The most pronounced effect of MAP during the entire storage was measured following the FRAP assay. Moreover, after 20 days of storage, the antioxidant capacity was about 63% higher in MAP samples than in ambient conditions, when measured by FRAP. Differences in results between methods may be explained by their different operating principles, as well as by the varying responses of antioxidants and oxidizing substances to different radicals or metal ions. Regardless of the method used, all findings strongly suggest that active MAP preserves antioxidant capacity more effectively than ambient storage which is consistent with results obtained for some SMs. Precisely, the highest antioxidant capacity was recorded in leaves packaged in MAP after 17 days of storage, while in the same sample the highest content of caffeoylmalic and chlorogenic acids, TPC and TChl was noted according to all three methods. Also, it is evident that during the storage period AsA was significantly higher in MAP samples compared to the corresponding C samples, which coincides with the results for antioxidant capacity. A similar pattern was observed for phenolic compounds, which recorded higher values in MAP samples after 17 and 20 days. Antioxidants such as phenolic compounds and AsA are very sensitive to oxidation, and reduced O_2_ levels limit oxidative reactions resulting in higher stability of these compounds, as previously discussed. Also, limited availability of O_2_ in MAP is associated with reduced respiration rate in plant tissues which can help preservation of antioxidants [14,50]. On the other hand, increased CO_2_ levels have been reported to be associated with reduced enzymatic activity and oxidative degradation in plant tissues [5,79]. Overall, the obtained results show that MAP was associated with higher antioxidant capacity in nettle leaves, in agreement with previous findings on leafy vegetables [34,46,97].

The effect of moisture absorbers in MAP during storage on antioxidant capacity is presented in Figure 6b, and the statistical significance of the tested factors in Table S3b. Results showed that the moisture absorbers combined with days of storage (A × D) had a great impact on antioxidant capacity (*p* = 0.0140 for ABTS, *p* ≤ 0.0001 for DPPH and FRAP). Antioxidant capacity values in MAP+A samples were generally significantly lower or statistically the same as those in MAP samples. According to the ABTS and FRAP methods, on days 14 and 20 there was no statistical difference between MAP and MAP+A samples, but on day 17 antioxidant capacity levels were higher in MAP packages. The largest difference in results was observed with the FRAP method on day 17, when antioxidant capacity dropped sharply by 27% in MAP+A packages, compared to MAP. The negative impact of absorbers was recorded throughout the entire period according to the DPPH method, with a statistically lower antioxidant capacity in leaves packaged in MAP+A compared to MAP. Across all three antioxidant assays, the highest values were observed after 17 days in MAP samples (without moisture absorbers), which also exhibited the greatest levels of TPC, caffeoylmalic acid, TChl, and TCar. DPPH results were consistent with TPC and caffeoylmalic acid, showing higher values in MAP samples throughout storage, while ABTS and FRAP followed trends similar to TChl, with peak values at day 17 and no significant differences between treatments at days 14 and 20. Data from the literature suggests that moisture levels play a complex role in antioxidant stability: high water content may dilute antioxidants, whereas excessive dehydration can damage cellular structures, leading to osmotic stress, membrane disruption, enzyme release, and accelerated oxidative degradation of antioxidant compounds [106,107,108,109,110,111,112]. In this study, moisture removal induced by silica gel sachets may be too intense, causing dehydration in plant tissue. In contrast to the beneficial effects of modified atmosphere packaging (MAP) alone, the addition of moisture absorbers generally had neutral to negative effects on antioxidant capacity. These findings indicate that, although MAP is an effective strategy for preserving the antioxidant capacity of nettle leaves, moisture regulation must be carefully optimized, as excessive water loss may compromise antioxidant stability.

## 4. Conclusions

This study evaluated the effectiveness of modified atmosphere packaging (MAP), alone and in combination with moisture-absorbing material, in preserving selected specific metabolites in nettle leaves during extended storage to promote their utilization and commercialization as a novel leafy vegetable. The findings of this study clearly demonstrate that MAP effectively preserved key specific metabolites (SMs) in nettle leaves compared to ambient atmosphere packaging. Precisely, major individual phenolic compounds (caffeoylmalic and chlorogenic acid), total phenolic compounds (TPC), ascorbic acid (AsA), total chlorophylls (TChl) and antioxidant capacity were generally significantly higher in MAP-packages. These results underscore the potential of MAP as a valuable postharvest strategy for preserving the nutritional and functional properties of stinging nettle. Despite the demonstrated benefits of modified atmosphere, the addition of moisture absorbers did not consistently improve specific metabolite levels, as the content of caffeoylmalic and chlorogenic acid, TPC, AsA, TChl and antioxidant capacity mostly showed similar or lower values in the presence of absorbers. However, the obtained results are valuable because they show that it is not necessary to use absorbers in MAP for preservation of nettle’s SMs, which, from a practical perspective, ultimately simplifies, speeds up and reduces the cost of the packaging process while aligning with the principles of sustainable packaging by eliminating unnecessary materials. These findings provide insight into the behavior of nettle leaf specific metabolites under different packaging conditions during storage and may contribute to post-harvest science, provide new insights into optimizing storage strategies and support the integration of new leafy vegetables into the market. Further studies at the molecular level are needed to elucidate the mechanisms underlying these results.

## Data Availability

The original contributions presented in this study are included in the article/Supplementary Materials. Further inquiries can be directed to the corresponding author.

## Supplementary Materials

The following supporting information can be downloaded at: https://www.mdpi.com/article/10.3390/foods15101731/s1, Figure S1: Chromatograms (λ = 270 and 360 nm) of samples after 20 days of storage; Table S1: The gradient elution mode of mobile phases for HPLC analyses of phenolic compounds; Table S2: Chromatographic and calibration curve data of analyzed phenolic compounds; Table S3: Statistical significances for water content, specific metabolites and antioxidant capacity of packaged nettle leaves according to (a) type of initial atmosphere and storage days and (b) moisture absorber and storage days. Table S4. The content of additional individual phenolic compounds (mg/100 g fm) in fresh nettle leaves stored for 20 days and packaged in (a) modified and ambient atmosphere, (b) modified atmosphere with and without moisture absorbers.
