# Supplementary material for: Optimizing Gas Composition and Moisture Content for Preservation of Specific Metabolites of Cultivated Stinging Nettle (Urtica dioica L.) Leaves
Source: Foods. 2026 May 14;15(10):1731. doi: 10.3390/foods15101731 (PMC13206582; doi:10.3390/foods15101731)
Supplement: Supplementary file 1 [file foods-15-01731-s001.zip › foods-4243595-supplementary.docx]

Article

Optimizing Gas Composition and Moisture Content for
Preservation of Specfic Metabolites of Cultivated Stinging
Nettle (*Urtica dioica* L.) Leaves

Mia Dujmović ^1^, Mia Kurek ^2,^*, Sandra Voća ^1^, Nevena Opačić ^1^, Sanja Radman ^1^, Zdenko Mlinar ^3^, Jana Šic Žlabur ^1^

^1^ University of Zagreb Faculty of Agriculture, Svetošimunska cesta 25, Zagreb, Croatia

^2^ University of Zagreb Faculty of Food Technology and Biotechnology, Pierottijeva 6, Zagreb, Croatia

^3^ Teaching Institute of Public Health "Dr. Andrija Štampar", Mirogojska cesta 16, Zagreb, Croatia

***** Correspondence: mkurek@pbf.hr; Tel.: + 385 1 4605 003

Table S1. The gradient elution mode of mobile phases for HPLC analyses of phenolic compounds; A – H_2_O with 3% formic acid, B – ACN with 3% formic acid.

| Time (min) | A (%) | B (%) |
| --- | --- | --- |
| 0 | 90 | 10 |
| 25 | 60 | 40 |
| 30 | 30 | 70 |
| 35-45 | 90 | 10 |

Table S2. Chromatographic and calibration curve data of analyzed phenolic compounds

| Phenolic compound | Peak mark | Retention time (min) | Calibration curve equation | Coefficient of determination (R^2^) |
| --- | --- | --- | --- | --- |
| Phenolic acids | | | | |
| Caffeoylmalic acid | 3 | 12.5 | y = 20636.8x + 16807 | 0.9997 |
| Chlorogenic acid | 1 | 9.5 | y = 14511.7x – 66325.3 | 0.9998 |
| Vanillic acid | 2 | 10.25 | y = 65980.1x + 143637 | 0.9999 |
| Flavonoids | | | | |
| Naringin | 4 | 16.3 | y = 3941.28x - 30329.7 | 0.9830 |

a)


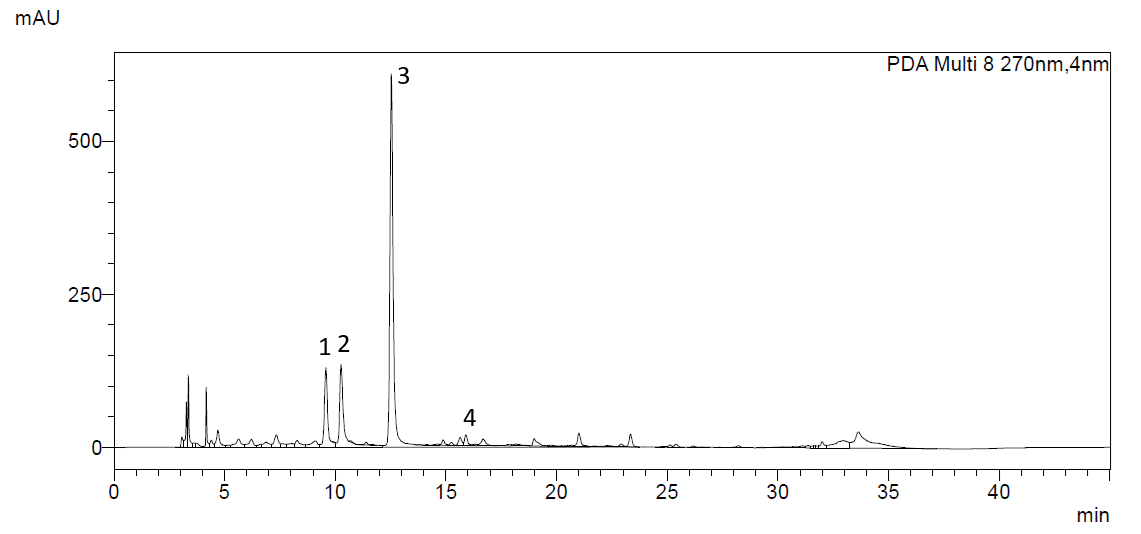


b)


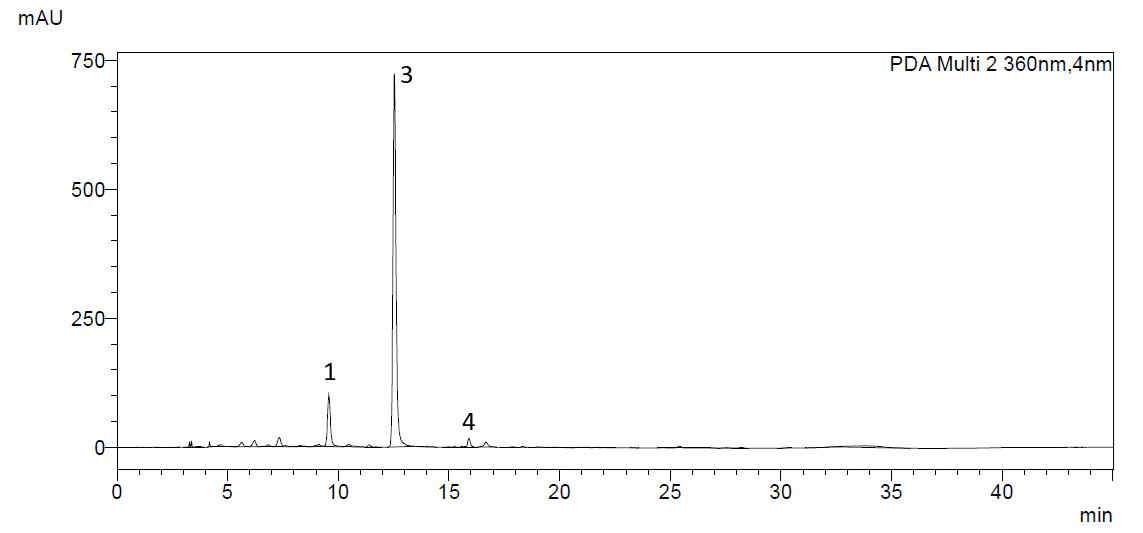


c)


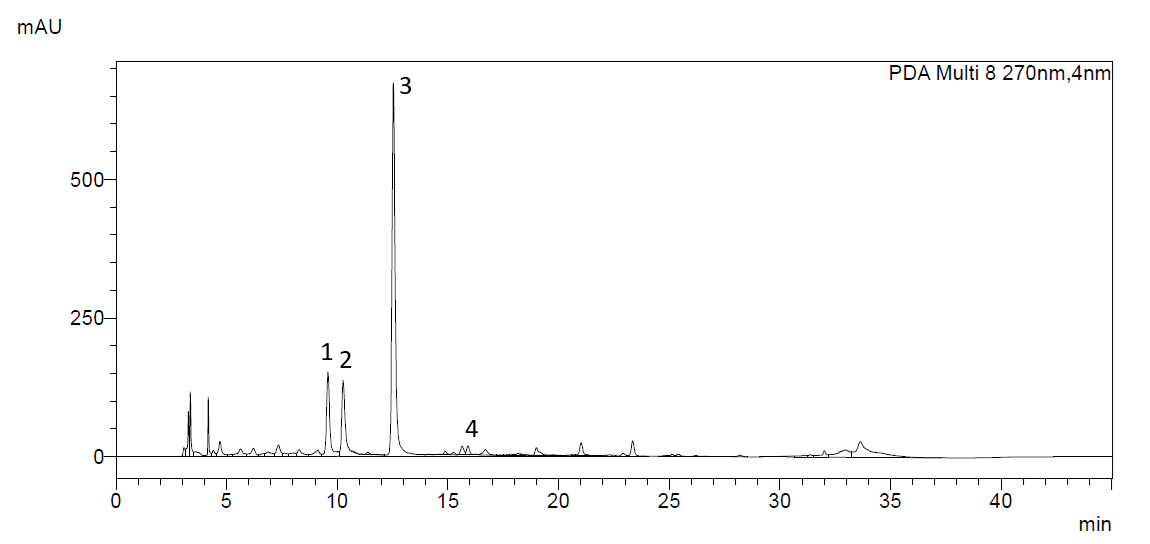


d)


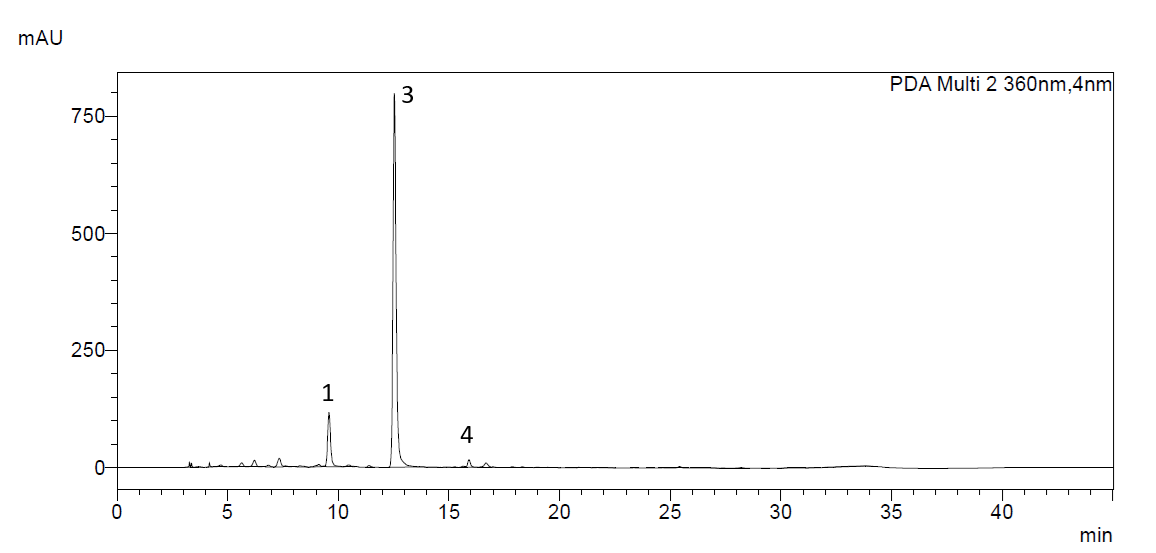


e)


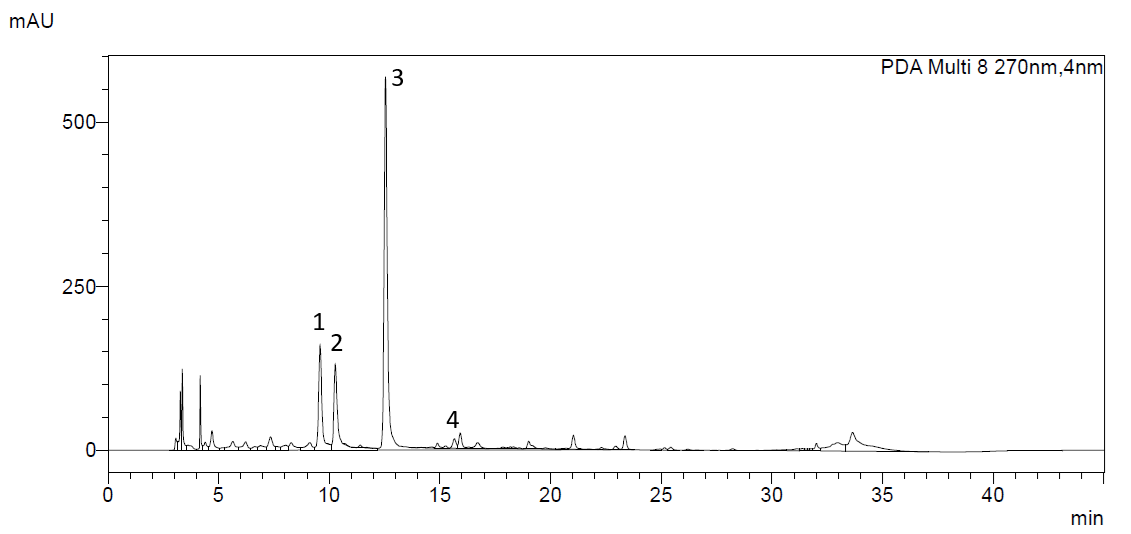


f)


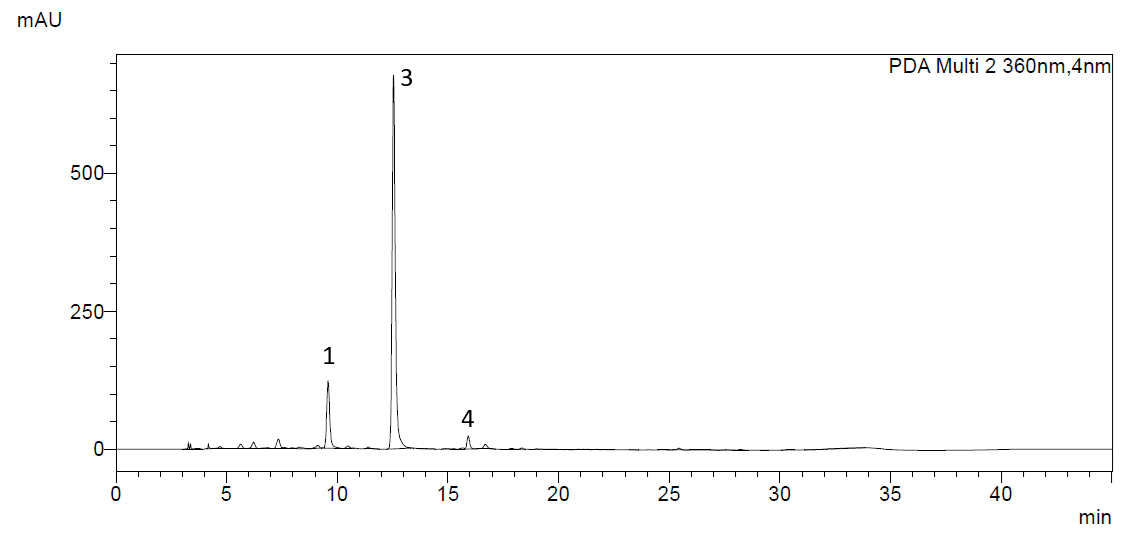


Figure S1. Chromatograms (λ = 270 and 360 nm) of samples after 20 days of storage; (a) and (b) nettle leaves packed in ambient atmosphere; (c) and (d) nettle leaves packed in modified atmosphere; (e) and (f) nettle leaves packed in modified atmosphere with moisture absorbers; 1 – chlorogenic acid, 2 – vanillic acid, 3 – caffeoylmalic acid, 4 – naringin.

Table S3. Statistical significances for water content, specific metabolites and antioxidant capacity of packaged nettle leaves according to (a) type of initial atmosphere and storage days and (b) moisture absorber and storage days; tested factors: AT – atmosphere type, D – days of storage, AT x D – interaction of atmosphere type and days of storage, A – presence of moisture absorber, A x D – interaction of moisture absorber and days of storage.

a)

| Dependent variables | ANOVA | LSD | AT x D | AT | D |
| --- | --- | --- | --- | --- | --- |
| Water content | 0.0008 | 0.7523 | 0.0025 | 0.0016 | 0.0004 |
| Caffeoylmalic acid | ≤0.0001 | 1.6982 | ≤0.0001 | ≤0.0001 | ≤0.0001 |
| Chlorogenic acid | ≤0.0001 | 2.8962 | ≤0.0001 | ≤0.0001 | ≤0.0001 |
| Vanillic acid | ≤0.0001 | 0.9885 | ≤0.0001 | 0.6173 | ≤0.0001 |
| Naringin | ≤0.0001 | 2.0741 | 0.0474 | 0.0389 | ≤0.0001 |
| Total phenolic compounds | ≤0.0001 | 2.2148 | ≤0.0001 | ≤0.0001 | ≤0.0001 |
| Total flavonoid compounds | ≤0.0001 | 3.3512 | ≤0.0001 | ≤0.0001 | ≤0.0001 |
| Total non-flavonoid compounds | ≤0.0001 | 2.698 | ≤0.0001 | ≤0.0001 | ≤0.0001 |
| Ascorbic acid | ≤0.0001 | 2.3536 | 0.0022 | ≤0.0001 | ≤0.0001 |
| Chlorophyll a | ≤0.0001 | 0.0092 | ≤0.0001 | ≤0.0001 | ≤0.0001 |
| Chlorophyll b | ≤0.0001 | 0.0175 | 0.0901 | 0.0001 | ≤0.0001 |
| Total chlorophyll content | ≤0.0001 | 0.0218 | ≤0.0001 | ≤0.0001 | ≤0.0001 |
| Total carotenoid content | ≤0.0001 | 0.0011 | ≤0.0001 | ≤0.0001 | ≤0.0001 |
| ABTS | ≤0.0001 | 0.1811 | ≤0.0001 | ≤0.0001 | ≤0.0001 |
| DPPH | ≤0.0001 | 0.069 | ≤0.0001 | ≤0.0001 | ≤0.0001 |
| FRAP | ≤0.0001 | 1.9751 | 0.0024 | ≤0.0001 | 0.0003 |

b)

| Dependent variables | ANOVA | LSD | A x D | A | D |
| --- | --- | --- | --- | --- | --- |
| Water content | ≤0.0001 | 0.986 | 0.5726 | ≤0.0001 | 0.0002 |
| Caffeoylmalic acid | ≤0.0001 | 1.0864 | ≤0.0001 | ≤0.0001 | ≤0.0001 |
| Chlorogenic acid | ≤0.0001 | 2.7185 | 0.0004 | ≤0.0001 | 0.0013 |
| Vanillic acid | ≤0.0001 | 1.0034 | ≤0.0001 | ≤0.0001 | ≤0.0001 |
| Naringin | ≤0.0001 | 1.5672 | ≤0.0001 | ≤0.0001 | ≤0.0001 |
| Total phenolic compounds | ≤0.0001 | 1.2459 | ≤0.0001 | ≤0.0001 | ≤0.0001 |
| Total flavonoid compounds | ≤0.0001 | 3.6411 | ≤0.0001 | ≤0.0001 | ≤0.0001 |
| Total non-flavonoid compounds | ≤0.0001 | 3.4255 | 0.0033 | 0.0026 | ≤0.0001 |
| Ascorbic acid | ≤0.0001 | 2.2486 | ≤0.0001 | ≤0.0001 | ≤0.0001 |
| Chlorophyll a | ≤0.0001 | 0.0092 | ≤0.0001 | 0.0005 | ≤0.0001 |
| Chlorophyll b | 0.0003 | 0.0175 | 0.0021 | 0.0075 | ≤0.0001 |
| Total chlorophyll content | 0.0009 | 0.039 | ≤0.0001 | 0.0043 | 0.5395 |
| Total carotenoid content | ≤0.0001 | 0.001 | ≤0.0001 | ≤0.0001 | ≤0.0001 |
| ABTS | ≤0.0001 | 0.1546 | 0.0140 | 0.0060 | ≤0.0001 |
| DPPH | ≤0.0001 | 0.0998 | ≤0.0001 | ≤0.0001 | ≤0.0001 |
| FRAP | ≤0.0001 | 2.0175 | ≤0.0001 | ≤0.0001 | 0.0002 |

Table S4. The content of additional individual phenolic compounds (mg/100 g fm) in fresh nettle leaves stored for 20 days and packaged in (a) modified and ambient atmosphere, (b) modified atmosphere with and without moisture absorbers. Means ± SD followed by the same letters are not significantly different at *p* ≤ 0.05 by LSD test (n = 3). Samples: C – leaves packaged in ambient atmosphere with no gas modification, MAP – leaves packaged with active modified atmosphere (5% O_2_/5%CO_2_), MAP+A – leaves packaged with active modified atmosphere and moisture absorber; tested factors: AT – atmosphere type, D – days of storage, AT x D – interaction of atmosphere type and days of storage, A – presence of moisture absorber, A x D – interaction of moisture absorber and days of storage.

| a) | | Caffeic acid | Coumaric acid | Ellagic acid | Ferulic acid | Gallic acid | Protocatehuic acid | 4-hydroxybenzoic acid | Kaempferol | Quercetin | Quercetin-3-glucoside | Rutin trihydrate |
| --- | --- | --- | --- | --- | --- | --- | --- | --- | --- | --- | --- | --- |
| 14 days of storage | MAP | 3.11±0.01^ab^ | 0.36±0.03^c^ | 0.34±0.03^a^ | 3.86±0.01^c^ | 3.46±0.03^bc^ | 10.26±0.1^a^ | 0.88±0.01^ab^ | 11.91±0.01^c^ | 3.38±0.01^b^ | 7.17±0.01d | 7.12±0.01^ab^ |
|  | C | 1.64±0.02^d^ | 0.46±0.01^bc^ | 0.35±0.01^a^ | 9.38±0.01^ab^ | 3.52±0.03^abc^ | 11.10±0.59^a^ | 0.93±0.01^a^ | 12.00±0.01^b^ | 3.38±0.01^b^ | 7.17±0.01^d^ | 7.10±0.01^abc^ |
| 17 days of storage | MAP | 3.30±0.02^a^ | 0.61±0.25^b^ | 0.26±0.01^b^ | 10.16±0.01^a^ | 3.47±0.02^bc^ | 10.96±0.83^a^ | 0.89±0.01^ab^ | 12.01±0.01^b^ | 3.38±0.01^b^ | 10.72±0.01^a^ | 7.14±0.01^a^ |
|  | C | 2.75±0.02^c^ | 0.89±0.03^a^ | 0.26±0.01^b^ | 9.12±0.25^b^ | 3.69±0.01^a^ | 10.50±0.21^a^ | 0.87±0.01^b^ | 12.04±0.01^ab^ | 3.39±0.01^a^ | 10.27±0.01^b^ | 7.05±0.01^c^ |
| 20 days of storage | MAP | 2.87±0.46^bc^ | 0.92±0.05^a^ | 0.30±0.08^ab^ | 9.00±1.21^b^ | 3.42±0.3^c^ | 10.11±1.81^a^ | 0.85±0.08^b^ | 12.01±0.12^b^ | 3.39±0.01^a^ | 10.09±0.01^bc^ | 7.07±0.05^c^ |
|  | C | 1.80±0.09^d^ | 0.38±0.02^c^ | 0.27±0.01^b^ | 8.59±0.02^b^ | 3.64±0.01^ab^ | 10.46±0.26^a^ | 0.88±0.01^ab^ | 12.10±0.01^a^ | 3.39±0.01^a^ | 9.85±0.01^c^ | 7.08±0.01^bc^ |
| ANOVA | | ≤0.0001 | 0.0002 | 0.0441 | ≤0.0001 | 0.1221 | 0.6345 | 0.2324 | 0.0273 | 0.0015 | ≤0.0001 | 0.0232 |
| LSD | | 0.347 | 0.1828 | 0.0605 | 0.889 | 0.217 | 1.5532 | 0.0556 | 0.0876 | 0.0054 | 0.4011 | 0.0454 |
| Statistical significance of varied factors | | | | | | | | | | | | |
| AT x D | | 0.0126 | ≤0.0001 | 0.5972 | ≤0.0001 | 0.4816 | 0.4399 | 0.2766 | 0.4813 | 0.2931 | 0.2779 | 0.1385 |
| AT | | 0.7579 | 0.3126 | 0.6364 | 0.0002 | 0.0211 | 0.5587 | 0.2568 | 0.0109 | 0.0671 | 0.0588 | 0.8028 |
| D | | ≤0.0001 | 0.0006 | 0.0041 | ≤0.0001 | 0.4992 | 0.6260 | 0.1432 | 0.0130 | 0.0004 | ≤0.0001 | 0.0081 |

| b) | | Caffeic acid | Coumaric acid | Ellagic acid | Ferulic acid | Gallic acid | Protocatehuic acid | 4-hydroxybenzoic acid | Kaempferol | Quercetin | Quercetin-3-glucoside | Rutin trihydrate |  |
| --- | --- | --- | --- | --- | --- | --- | --- | --- | --- | --- | --- | --- | --- |
| 14 days of storage | MAP | 1.80±0.09^b^ | 0.36±0.03^c^ | 0.34±0.03^a^ | 3.86±0.01^d^ | 3.46±0.03^b^ | 10.26±0.1^a^ | 0.88±0.01^ab^ | 11.91±0.01^c^ | 3.38±0.01^c^ | 10.72±0.01^b^ | 7.12±0.01^ab^ |  |
|  | MAP+A | 1.83±0.01^b^ | 0.26±0.06^c^ | 0.34±0.01^a^ | 3.96±0.01^d^ | 3.39±0.01^b^ | 10.29±0.1^a^ | 0.80±0.01^c^ | 11.96±0.02^bc^ | 3.38±0.01^c^ | 7.21±0.01^d^ | 7.16±0.01^a^ |  |
| 17 days of storage | MAP | 3.11±0.01^a^ | 0.61±0.25^b^ | 0.26±0.01^b^ | 10.16±0.01^ab^ | 3.47±0.02^b^ | 10.96±0.83^a^ | 0.89±0.01^ab^ | 12.01±0.01^ab^ | 3.38±0.01^bc^ | 11.18±0.01^a^ | 7.10±0.01^bc^ |  |
|  | MAP+A | 2.78±0.01^a^ | 0.37±0.03^c^ | 0.27±0.01^b^ | 9.60±0.01^bc^ | 3.60±0.02^b^ | 7.62±0.07^b^ | 0.87±0.01^ab^ | 12.03±0.01^ab^ | 3.39±0.01^b^ | 10.43±0.01^bc^ | 7.07±0.01^c^ |  |
| 20 days of storage | MAP | 3.09±0.01^a^ | 0.92±0.05^a^ | 0.30±0.08^ab^ | 10.94±0.01^a^ | 3.42±0.3^b^ | 10.11±1.81^a^ | 0.85±0.08^bc^ | 12.07±0.01^a^ | 3.39±0.01^b^ | 10.09±0.54^c^ | 7.07±0.05^c^ |  |
|  | MAP+A | 2.87±0.46^a^ | 0.42±0.04^bc^ | 0.27±0.01^b^ | 9.00±1.21^c^ | 3.87±0.04^a^ | 8.38±0.01^b^ | 0.93±0.01^a^ | 12.01±0.12^ab^ | 3.40±0.01^a^ | 7.17±0.01^d^ | 7.05±0.01^c^ |  |
| ANOVA | | ≤0.0001 | 0.0007 | 0.0720 | ≤0.0001 | 0.0100 | 0.0024 | 0.0190 | 0.0378 | 0.0028 | ≤0.0001 | 0.0110 |  |
| LSD | | 0.3439 | 0.2028 | 0.0603 | 0.8965 | 0.2192 | 1.3186 | 0.0567 | 0.086 | 0.0079 | 0.4007 | 0.0476 |  |
| Statistical significance of varied factors | | | | | | | | | | | | | |
| A x D | | 0.0817 | 0.0292 | 0.5616 | 0.0041 | 0.0168 | 0.0113 | 0.0040 | 0.7791 | 0.2351 | 0.0008 | 0.0850 |  |
| A | | 0.7857 | 0.0003 | 0.5865 | 0.0678 | 0.0203 | 0.0008 | 0.6661 | 0.0969 | 0.0333 | 0.0267 | 0.9265 |  |
| D | | ≤0.0001 | 0.0007 | 0.0084 | ≤0.0001 | 0.0424 | 0.0765 | 0.0485 | 0.0064 | 0.0008 | ≤0.0001 | 0.0006 |  |
