# Supplementary material for: Optimizing Gas Composition and Moisture Content for Preservation of Specific Metabolites of Cultivated Stinging Nettle (Urtica dioica L.) Leaves
Source: Foods. 2026 May 14;15(10):1731. doi: 10.3390/foods15101731 (PMC13206582; doi:10.3390/foods15101731)
Supplement: Supplementary file 1 [file foods-15-01731-s001.zip › foods-4243595-supplementary.pdf]

Article

Optimizing Gas Composition and Moisture Content for Preservation of Specfic Metabolites of Cultivated Stinging Nettle (*Urtica dioica* L.) Leaves

Mia Dujmović [1], Mia Kurek [2,*], Sandra Voća [1], Nevena Opačić [1], Sanja Radman [1], Zdenko Mlinar [3], Jana Šic Žlabur [1]

Table S1. The gradient elution mode of mobile phases for HPLC analyses of phenolic compounds; A – H_2O with 3% formic acid, B – ACN with 3% formic acid.

Time (min)	A (%)	B (%)
0	90	10
25	60	40
30	30	70
35-45	90	10

Table S2. Chromatographic and calibration curve data of analyzed phenolic compounds

Phenolic compound	Peak mark	Retention time (min)	Calibration curve equation	Coefficient of determination (R^2)
Phenolic acids				
Caffeoylmalic acid	3	12.5	y = 20636.8x + 16807	0.9997
Chlorogenic acid	1	9.5	y = 14511.7x – 66325.3	0.9998
Vanillic acid	2	10.25	y = 65980.1x + 143637	0.9999
Flavonoids				
Naringin	4	16.3	y = 3941.28x - 30329.7	0.9830

a)

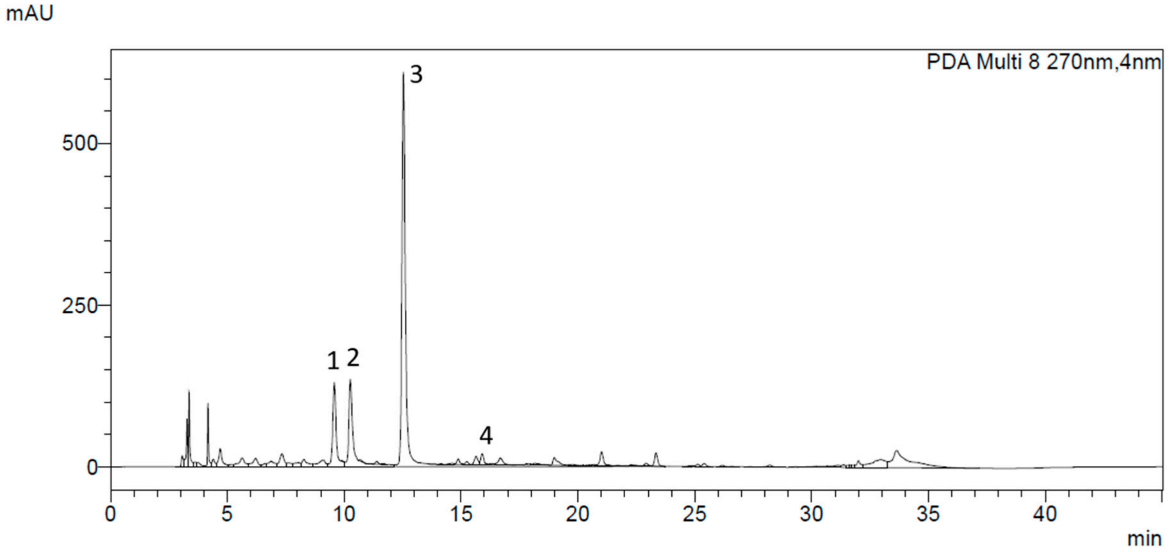
mAU
PDA Multi 8 270nm,4nm
500
250
0
1
2
3
4
0
5
10
15
20
25
30
35
40
min

b)

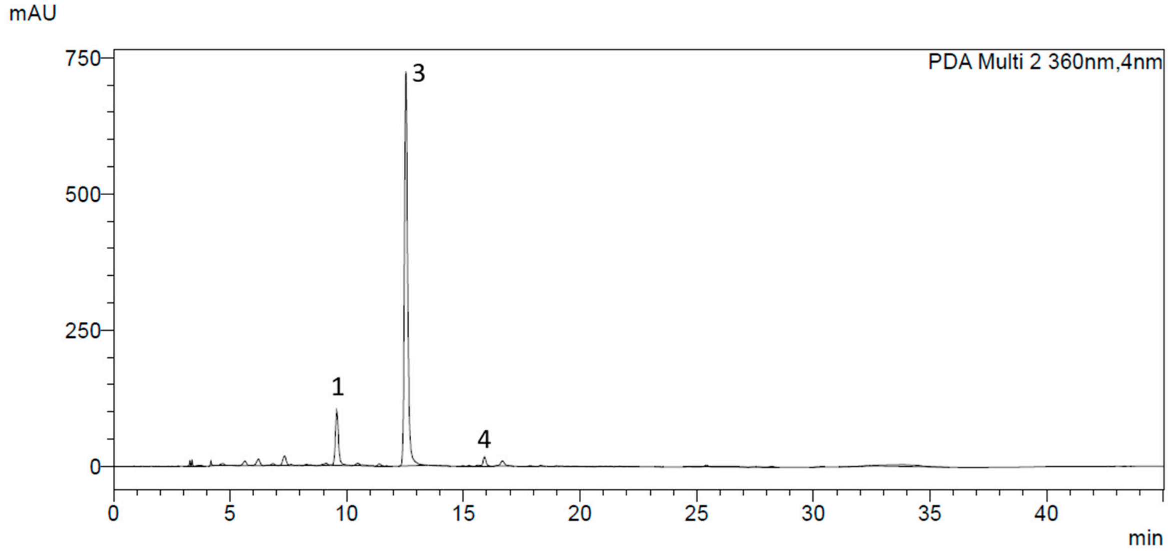
mAU
PDA Multi 2 360nm,4nm
750
500
250
0
1
3
4
0
5
10
15
20
25
30
35
40
min

c)

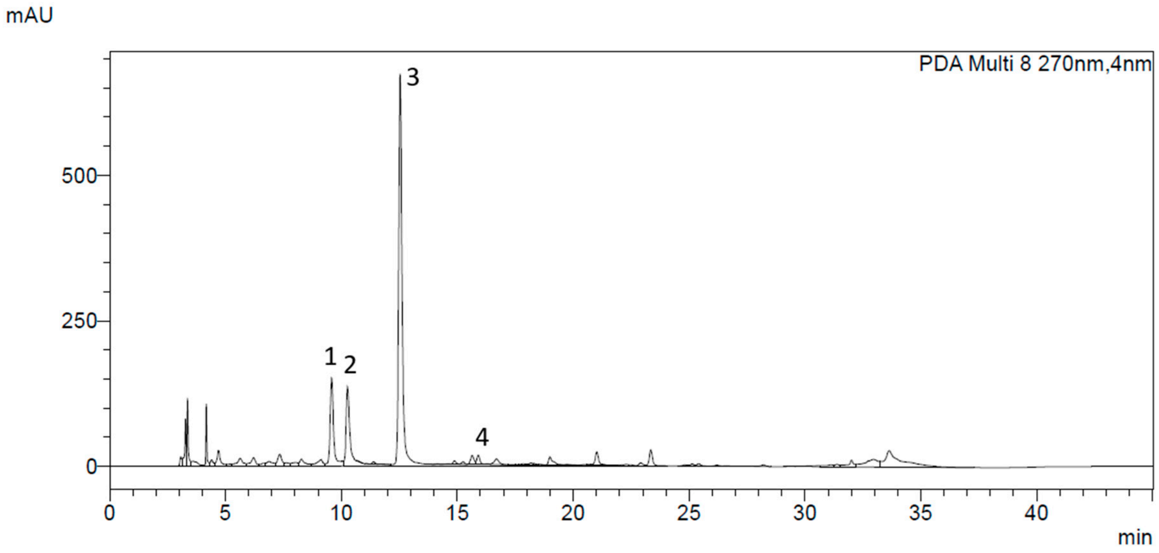

mAU
PDA Multi 8 270nm,4nm
500
250
0
1
2
3
4
0
5
10
15
20
25
30
35
40
min

d)

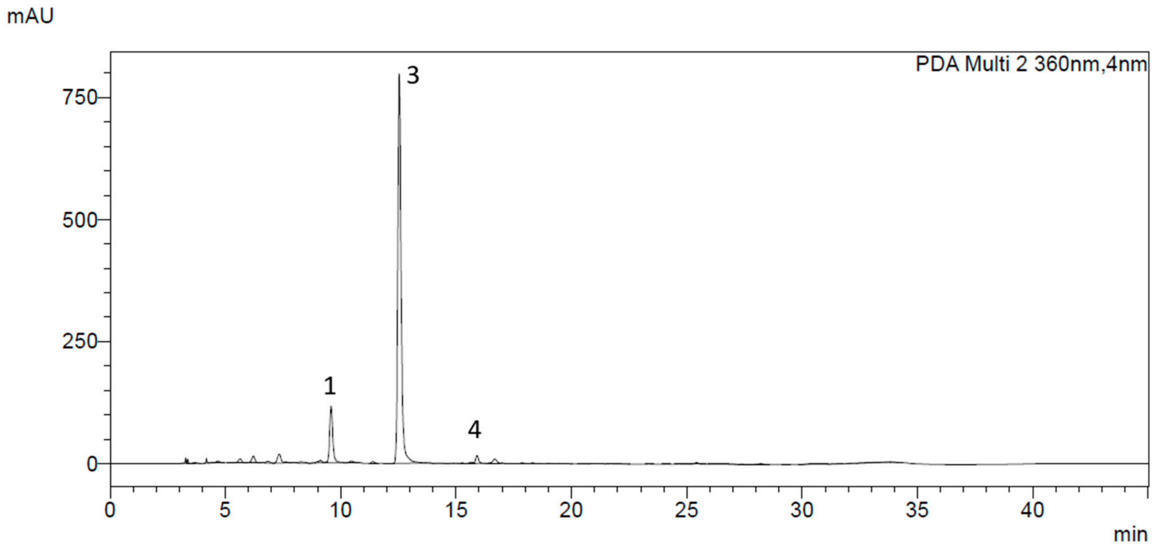

mAU
PDA Multi 2 360nm,4nm
750
500
250
0
1
3
4
0
5
10
15
20
25
30
35
40
min

e)

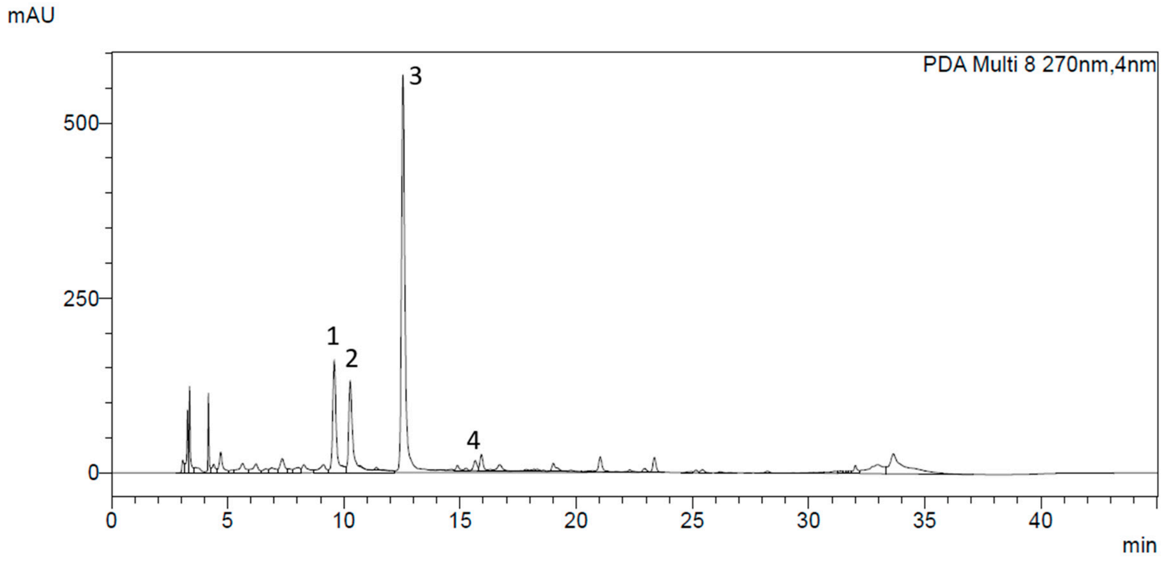

f)

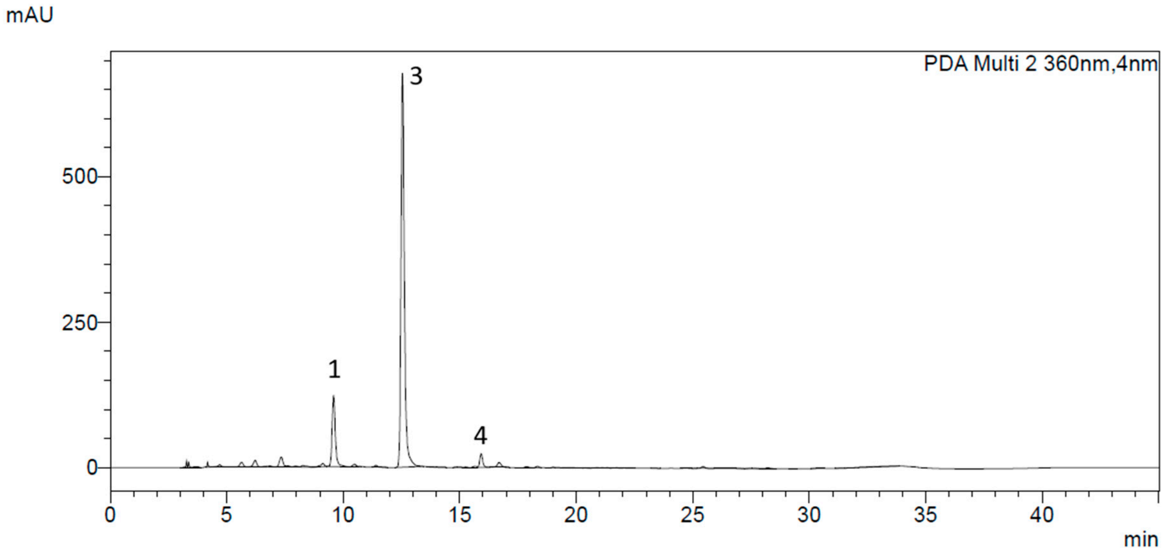

Figure S1. Chromatograms (λ = 270 and 360 nm) of samples after 20 days of storage; (a) and (b) nettle leaves packed in ambient atmosphere; (c) and (d) nettle leaves packed in modified atmosphere; (e) and (f) nettle leaves packed in modified atmosphere with moisture absorbers; 1 – chlorogenic acid, 2 – vanillic acid, 3 – caffeoylmalic acid, 4 – naringin.

Table S3. Statistical significances for water content, specific metabolites and antioxidant capacity of packaged nettle leaves according to (a) type of initial atmosphere and storage days and (b) moisture absorber and storage days; tested factors: AT – atmosphere type, D – days of storage, AT x D – interaction of atmosphere type and days of storage, A – presence of moisture absorber, A x D – interaction of moisture absorber and days of storage.

a)

Dependent variables	ANOVA	LSD	AT x D	AT	D
Water content	0.0008	0.7523	0.0025	0.0016	0.0004
Caffeoylmalic acid	≤0.0001	1.6982	≤0.0001	≤0.0001	≤0.0001
Chlorogenic acid	≤0.0001	2.8962	≤0.0001	≤0.0001	≤0.0001
Vanillic acid	≤0.0001	0.9885	≤0.0001	0.6173	≤0.0001
Naringin	≤0.0001	2.0741	0.0474	0.0389	≤0.0001
Total phenolic compounds	≤0.0001	2.2148	≤0.0001	≤0.0001	≤0.0001
Total flavonoid compounds	≤0.0001	3.3512	≤0.0001	≤0.0001	≤0.0001
Total non-flavonoid compounds	≤0.0001	2.698	≤0.0001	≤0.0001	≤0.0001
Ascorbic acid	≤0.0001	2.3536	0.0022	≤0.0001	≤0.0001
Chlorophyll a	≤0.0001	0.0092	≤0.0001	≤0.0001	≤0.0001
Chlorophyll b	≤0.0001	0.0175	0.0901	0.0001	≤0.0001
Total chlorophyll content	≤0.0001	0.0218	≤0.0001	≤0.0001	≤0.0001
Total carotenoid content	≤0.0001	0.0011	≤0.0001	≤0.0001	≤0.0001
ABTS	≤0.0001	0.1811	≤0.0001	≤0.0001	≤0.0001
DPPH	≤0.0001	0.069	≤0.0001	≤0.0001	≤0.0001
FRAP	≤0.0001	1.9751	0.0024	≤0.0001	0.0003

b)

Dependent variables	ANOVA	LSD	A x D	A	D
Water content	≤0.0001	0.986	0.5726	≤0.0001	0.0002
Caffeoylmalic acid	≤0.0001	1.0864	≤0.0001	≤0.0001	≤0.0001
Chlorogenic acid	≤0.0001	2.7185	0.0004	≤0.0001	0.0013
Vanillic acid	≤0.0001	1.0034	≤0.0001	≤0.0001	≤0.0001
Naringin	≤0.0001	1.5672	≤0.0001	≤0.0001	≤0.0001
Total phenolic compounds	≤0.0001	1.2459	≤0.0001	≤0.0001	≤0.0001
Total flavonoid compounds	≤0.0001	3.6411	≤0.0001	≤0.0001	≤0.0001
Total non-flavonoid compounds	≤0.0001	3.4255	0.0033	0.0026	≤0.0001
Ascorbic acid	≤0.0001	2.2486	≤0.0001	≤0.0001	≤0.0001
Chlorophyll a	≤0.0001	0.0092	≤0.0001	0.0005	≤0.0001
Chlorophyll b	0.0003	0.0175	0.0021	0.0075	≤0.0001
Total chlorophyll content	0.0009	0.039	≤0.0001	0.0043	0.5395
Total carotenoid content	≤0.0001	0.001	≤0.0001	≤0.0001	≤0.0001
ABTS	≤0.0001	0.1546	0.0140	0.0060	≤0.0001
DPPH	≤0.0001	0.0998	≤0.0001	≤0.0001	≤0.0001
FRAP	≤0.0001	2.0175	≤0.0001	≤0.0001	0.0002

Table S4. The content of additional individual phenolic compounds (mg/100 g fm) in fresh nettle leaves stored for 20 days and packaged in (a) modified and ambient atmosphere, (b) modified atmosphere with and without moisture absorbers. Means ± SD followed by the same letters are not significantly different at $p \leq 0.05$ by LSD test (n = 3). Samples: C – leaves packaged in ambient atmosphere with no gas modification, MAP – leaves packaged with active modified atmosphere (5% O_2/5%CO_2), MAP+A – leaves packaged with active modified atmosphere and moisture absorber; tested factors: AT – atmosphere type, D – days of storage, AT x D – interaction of atmosphere type and days of storage, A – presence of moisture absorber, A x D – interaction of moisture absorber and days of storage.

a)		Caffeic acid	Coumaric acid	Ellagic acid	Ferulic acid	Gallic acid	Protocatehuic acid	4-hydroxybenzoic acid	Kaempferol	Quercetin	Quercetin-3-glucoside	Rutin trihydrate
14 days of storage	MAP	3.11±0.01^{ab}	0.36±0.03^{c}	0.34±0.03^{a}	3.86±0.01^{c}	3.46±0.03^{bc}	10.26±0.1^{a}	0.88±0.01^{ab}	11.91±0.01^{c}	3.38±0.01^{b}	7.17±0.01d	7.12±0.01^{ab}
	C	1.64±0.02^{d}	0.46±0.01^{bc}	0.35±0.01^{a}	9.38±0.01^{ab}	3.52±0.03^{abc}	11.10±0.59^{a}	0.93±0.01^{a}	12.00±0.01^{b}	3.38±0.01^{b}	7.17±0.01^{d}	7.10±0.01^{abc}
17 days of storage	MAP	3.30±0.02^{a}	0.61±0.25^{b}	0.26±0.01^{b}	10.16±0.01^{a}	3.47±0.02^{bc}	10.96±0.83^{a}	0.89±0.01^{ab}	12.01±0.01^{b}	3.38±0.01^{b}	10.72±0.01^{a}	7.14±0.01^{a}
	C	2.75±0.02^{c}	0.89±0.03^{a}	0.26±0.01^{b}	9.12±0.25^{b}	3.69±0.01^{a}	10.50±0.21^{a}	0.87±0.01^{b}	12.04±0.01^{ab}	3.39±0.01^{a}	10.27±0.01^{b}	7.05±0.01^{c}
20 days of storage	MAP	2.87±0.46^{bc}	0.92±0.05^{a}	0.30±0.08^{ab}	9.00±1.21^{b}	3.42±0.3^{c}	10.11±1.81^{a}	0.85±0.08^{b}	12.01±0.12^{b}	3.39±0.01^{a}	10.09±0.01^{bc}	7.07±0.05^{c}
	C	1.80±0.09^{d}	0.38±0.02^{c}	0.27±0.01^{b}	8.59±0.02^{b}	3.64±0.01^{ab}	10.46±0.26^{a}	0.88±0.01^{ab}	12.10±0.01^{a}	3.39±0.01^{a}	9.85±0.01^{c}	7.08±0.01^{bc}
ANOVA		≤0.0001	0.0002	0.0441	≤0.0001	0.1221	0.6345	0.2324	0.0273	0.0015	≤0.0001	0.0232
LSD		0.347	0.1828	0.0605	0.889	0.217	1.5532	0.0556	0.0876	0.0054	0.4011	0.0454
Statistical significance of varied factors												
AT x D		0.0126	≤0.0001	0.5972	≤0.0001	0.4816	0.4399	0.2766	0.4813	0.2931	0.2779	0.1385
AT		0.7579	0.3126	0.6364	0.0002	0.0211	0.5587	0.2568	0.0109	0.0671	0.0588	0.8028
D		≤0.0001	0.0006	0.0041	≤0.0001	0.4992	0.6260	0.1432	0.0130	0.0004	≤0.0001	0.0081

b)		Caffeic acid	Coumaric acid	Ellagic acid	Ferulic acid	Gallic acid	Protocatehuic acid	4-hydroxyben-zoic acid	Kaempferol	Quercetin	Quercetin-3-glucoside	Rutin tri-hydrate
14 days of stor-age	MAP	1.80±0.09^{b}	0.36±0.03^{c}	0.34±0.03^{a}	3.86±0.01^{d}	3.46±0.03^{b}	10.26±0.1^{a}	0.88±0.01^{ab}	11.91±0.01^{c}	3.38±0.01^{c}	10.72±0.01^{b}	7.12±0.01^{ab}
	MAP+A	1.83±0.01^{b}	0.26±0.06^{c}	0.34±0.01^{a}	3.96±0.01^{d}	3.39±0.01^{b}	10.29±0.1^{a}	0.80±0.01^{c}	11.96±0.02^{bc}	3.38±0.01^{c}	7.21±0.01^{d}	7.16±0.01^{a}
17 days of stor-age	MAP	3.11±0.01^{a}	0.61±0.25^{b}	0.26±0.01^{b}	10.16±0.01^{ab}	3.47±0.02^{b}	10.96±0.83^{a}	0.89±0.01^{ab}	12.01±0.01^{ab}	3.38±0.01^{bc}	11.18±0.01^{a}	7.10±0.01^{bc}
	MAP+A	2.78±0.01^{a}	0.37±0.03^{c}	0.27±0.01^{b}	9.60±0.01^{bc}	3.60±0.02^{b}	7.62±0.07^{b}	0.87±0.01^{ab}	12.03±0.01^{ab}	3.39±0.01^{b}	10.43±0.01^{bc}	7.07±0.01^{c}
20 days of stor-age	MAP	3.09±0.01^{a}	0.92±0.05^{a}	0.30±0.08^{ab}	10.94±0.01^{a}	3.42±0.3^{b}	10.11±1.81^{a}	0.85±0.08^{bc}	12.07±0.01^{a}	3.39±0.01^{b}	10.09±0.54^{c}	7.07±0.05^{c}
	MAP+A	2.87±0.46^{a}	0.42±0.04^{bc}	0.27±0.01^{b}	9.00±1.21^{c}	3.87±0.04^{a}	8.38±0.01^{b}	0.93±0.01^{a}	12.01±0.12^{ab}	3.40±0.01^{a}	7.17±0.01^{d}	7.05±0.01^{c}
ANOVA		≤0.0001	0.0007	0.0720	≤0.0001	0.0100	0.0024	0.0190	0.0378	0.0028	≤0.0001	0.0110
LSD		0.3439	0.2028	0.0603	0.8965	0.2192	1.3186	0.0567	0.086	0.0079	0.4007	0.0476
Statistical significance of varied factors												
A x D		0.0817	0.0292	0.5616	0.0041	0.0168	0.0113	0.0040	0.7791	0.2351	0.0008	0.0850
A		0.7857	0.0003	0.5865	0.0678	0.0203	0.0008	0.6661	0.0969	0.0333	0.0267	0.9265
D		≤0.0001	0.0007	0.0084	≤0.0001	0.0424	0.0765	0.0485	0.0064	0.0008	≤0.0001	0.0006
